# Supplementary material for: Emergence of highly pathogenic H5N2 and H7N1 influenza A viruses from low pathogenic precursors by serial passage in ovo
Source: PLoS One. 2020 Oct 8;15(10):e0240290. doi: 10.1371/journal.pone.0240290 (PMC7544131; doi:10.1371/journal.pone.0240290)
Supplement: S1 Table — Genes encoded within each segment are in italics; nt: nucleotides. (DOCX) [file pone.0240290.s001.docx]

S1 Table: Ion Torrent sequencing results and read coverage for the H5N2 genome

| **Passage no.** | **Total reads** | **Ave. read length** | **No. reads mapped to each reference genome segment [S]^a^ (percentage of total reads)** | | | | | | | |
| --- | --- | --- | --- | --- | --- | --- | --- | --- | --- | --- |
|  |  |  | **S1**  2,341 nt  ***PB2*** | **S2**  2,341 nt  ***PB1+PB1 F2*** | **S3**  2,233 nt  ***PA+PAX*** | **S4**  1,728 nt  ***HA*** | **S5**  1,565 nt  ***NP*** | **S6**  1,460 nt  ***NA*** | **S7**  1,027 nt  ***M1+M2e*** | **S8**  890 nt  ***NS1+NEP*** |
| **1** | 5,214,382 | 85 | 124,539 (2.39) | 100,921 (1.94) | 120,911 (2.32) | 150,346 (2.88) | 205,669 (3.94) | 108,338 (2.08) | 158,291 (3.04) | 82,438 (1.58) |
| **2** | 4,126,840 | 69 | 27,266 (0.66) | 13,116 (0.32) | 22,751 (0.55) | 11,102 (0.27) | 18,160 (0.44) | 11,675 (0.28) | 15,621 (0.38) | 8,113  (0.20) |
| **3** | 5,585,369 | 138 | 18,961 (0.34) | 20,174 (0.36) | 35,287 (0.63) | 26,624 (0.48) | 38,648 (0.69) | 27,042 (0.48) | 33,697 (0.60) | 18,509 (0.33) |
| **4** | 3,915,305 | 112 | 77,269 (1.97) | 80,570 (2.06) | 99,220 (2,53) | 84,648 (2.16) | 135,887 (3.47) | 96,962 (2.48) | 102,747 (2.62) | 52,723 (1.35) |
| **5** | 6,807,720 | 114 | 9,834 (0.14) | 14,938 (0.22) | 19,032 (0.28) | 12,647 (0.19) | 24,484 (0.36) | 15,561 (0.23) | 23,379 (0.34) | 10,702 (0.16) |
| **6** | 6,840,676 | 125 | 12,706 (0.19) | 12,360 (0.18) | 15,736 (0.23) | 12,382 (0.18) | 22,999 (0.34) | 13,107 (0.19) | 15,491 (0.23) | 10,702 (0.10) |
| **7** | 17,729,022 | 61 | 259,859 (1.47) | 269,312 (1.52) | 283,371 (1.60) | 169,416 (0.96) | 355,059 (2.00) | 238,598 (1.35) | 249,781 (1.41) | 119,115 (0.67) |
| **11** | 5,474,612 | 98 | 376,580 (6.88) | 356,486 (6.51) | 314,595 (5.75) | 318,540 (5.82) | 360,017 (6.58) | 255,189 (4.66) | 278,090 (5.08) | 139,759 (2.55) |
| **15** | 16,737,895 | 110 | 88,045 (0.53) | 102,233 (0.61) | 132,516 (0.79) | 100,225 (0.60) | 136,337 (0.81) | 98,459 (0.59) | 112,533 (0.67) | 78,507 (0.47) |
| **17** | 13,011,290 | 115 | 167,177 (1.28) | 190,857 (1.47) | 245,676 (1.89) | 211,391 (1.62) | 313,532 (2.41) | 196,166 (1.51) | 229,150 (1.76) | 143,580 (1.10) |

^a^Genes encoded within each segment are in italics; nt: nucleotides.
