# Supplementary material for: Emergence of highly pathogenic H5N2 and H7N1 influenza A viruses from low pathogenic precursors by serial passage in ovo
Source: PLoS One. 2020 Oct 8;15(10):e0240290. doi: 10.1371/journal.pone.0240290 (PMC7544131; doi:10.1371/journal.pone.0240290)
Supplement: S2 Table — Genes encoded within each segment are in italics; nt: nucleotides. (DOCX) [file pone.0240290.s002.docx]

S2 Table: Ion Torrent sequencing results and read coverage for the H7N1 genome

| **Passage no.** | **Total reads** | **Ave. read length** | **No. reads mapped to each reference genome segment [S]^a^ (percentage of total reads)** | | | | | | | |
| --- | --- | --- | --- | --- | --- | --- | --- | --- | --- | --- |
|  |  |  | **S1**  2,341 nt  ***PB2*** | **S2**  2,341 nt  ***PB1+PB1 F2*** | **S3**  2,233 nt  ***PA+PAX*** | **S4**  1,728 nt  ***HA*** | **S5**  1,565 nt  ***NP*** | **S6**  1,460 nt  ***NA*** | **S7**  1,027 nt  ***M1+M2e*** | **S8**  890 nt  ***NS1+NEP*** |
| **1** | 3,592,450 | 118 | 68,521 (1.91) | 85,043 (2.37) | 79,682  (2.22) | 107,505 (2.99) | 150,361 (4.19) | 52,535 (1.46) | 99,837 (2.78) | 83,591  (2.33) |
| **2** | 4,549,340 | 130 | 137,657 (3.03) | 141,637 (3.11) | 159,801 (3.51) | 222,382 (4.89) | 297,162 (6.53) | 112,220 (2.47) | 285,976 (6.29) | 165,725 (3.64) |
| **3** | 10,283,029 | 129 | 182,632 (1.78) | 252,302 (2.45) | 215,822 (2.10) | 239,217 (2.33) | 304,316 (2.96) | 137,951 (1.34) | 242,194 (2.36) | 174,080 (1.69) |
| **4** | 4,720,300 | 91 | 2,569  (0.05) | 2,196  (0.05) | 3,100  (0.07) | 3,564  (0.08) | 6,210  (0.13) | 1,006  (0.02) | 6,164  (0.13) | 3,193  (0.07) |
| **5** | 6,971,338 | 117 | 6,421  (0.09) | 8,721  (0.13) | 11,571  (0.17) | 10,686 (0.15) | 21,409 (0.31) | 5,804  (0.08) | 15,109 (0.22) | 14,057  (0.20) |
| **6** | 4,852,060 | 131 | 60,100 (1.24) | 73,074 (1.51) | 70,022  (1.44) | 66,966 (1.38) | 61,027 (1.26) | 27,006 (0.56) | 42,213 (0.87) | 33,079  (0.68) |
| **7** | 19,361,277 | 119 | 205,284 (1.06) | 263,274 (1.36) | 292,098 (1.51) | 253,494 (1.31) | 224,818 (1.16) | 109,573 (0.57) | 148,205 (0.77) | 127,714 (0.66) |
| **11** | 5,534,804 | 141 | 356,525 (6.44) | 341,873 (6.18) | 297,547 (5.38) | 276,576 (5.00) | 311,570 (5.63) | 255,189 (2.96) | 191,716 (3.46) | 138,649 (2.51) |
| **15** | 16,322,496 | 116 | 462,692 (2.83) | 579,378 (3.55) | 482,100 (2.95) | 440,638 (2.70) | 513,903 (3.15) | 280,232 (1.72) | 526,100 (3.22) | 270,717 (1.66) |
| **17** | 11,562,133 | 114 | 200,544 (1.73) | 249,894 (2.16) | 214,138 (1.85) | 192,484 (1.66) | 229,679 (1.99) | 128,378 (1.11) | 268,125 (2.32) | 122,150 (1.06) |

**^a^**Genes encoded within each segment are in italics; nt: nucleotides.
