# Supplementary material for: Emergence of highly pathogenic H5N2 and H7N1 influenza A viruses from low pathogenic precursors by serial passage in ovo
Source: PLoS One. 2020 Oct 8;15(10):e0240290. doi: 10.1371/journal.pone.0240290 (PMC7544131; doi:10.1371/journal.pone.0240290)
Supplement: S3 Table — #Insertions, deletions and substitutions in relation to the conventional low pathogenic sequence‡ are underlined; stop codons are indicated by “*”. †In-frame HA2 [] total number of variants: total number of HACS reads ratio. (DOCX) [file pone.0240290.s003.docx]

**S3 Table: Variants detected at the hemagglutinin cleavage site (HA_0_) of H5N2 low pathogenic avian influenza viruses passaged in 14-day old embryonated chicken eggs**

1. **H5N2 Passage 1**

| **HA_0_ cDNA nucleotide sequence^#^** | **Main mutational**  **effect** | **Translated**  **amino acids** | **No. of reads**  **(percentage)** |
| --- | --- | --- | --- |
| **CCTCAAAGAGAGACAAGAGGGCTATTT**^‡^ |  | **PQRETRGLF**^†^ | 184 (82.14) |
| **CCTCAA_GAGAGACA__GAGGCTATTT** | codon deletion | **PQERQRLF**^†^ | 1 (0.44) |
| **CCTCAAAGAGGGACAAGAGGGCTATTT** | substitution | **PQRGTRGLF**^†^ | 2 (0.89) |
| **CCTCAAAGAGAGACAAGAGGGCTAGTT** | substitution | **PQRETRGLV**^†^ | 2 (0.89) |
| **CCTCAAAGACGAGACA_GAGGGCTATTT** | substitution | **PQRRDRGLF**^†^ | 1 (0.44) |
| **CCTCAAAGAGAGACAAGAGGGCCTATTT** | +1 frameshift | **PQRETRGPI** | 1 (0.44) |
| **CCTCCAAAGAGAGACAAGAGGGCTATTT** | +1 frameshift | **PPKRDKRAI** | 3 (1.09) |
| **CCTCCAAAGAGAGACAAGAGGGCTACTT** | +1 frameshift | **PPKRDKRAT** | 1 (0.44) |
| **CCTCAAGAGATGAACAAGAGGGCTATTT** | +1 frameshift | **PQEMNKRAI** | 1 (0.44) |
| **CCTCAAAGAGAGACAAGAGGGCTTATTT** | +1 frameshift | **PQRETRGLI** | 2 (0.89) |
| **CCTCAAAGAGAGGACAAGAGGGCTATTT** | +1 frameshift | **PQREDKRAI** | 1 (0.44) |
| **CCTCAAAGAGAGCACAAGAGGGCTATTT** | +1 frameshift | **PQREHKRAI** | 1 (0.44) |
| **CCTCAAAGAGAGACAATGAGGGCTATTT** | +1 frameshift | **PQRETMRAI** | 1 (0.44) |
| **CCTCAAAGAGAGACAAGAGGGGCTATTT** | +1 frameshift | **PQRETRGAI** | 1 (0.44) |
| **CCTCAAAGAGAGACAAGAAGGGCTATTT** | +1 frameshift | **PQRETRRAI** | 1 (0.44) |
| **CCTCAAAGAGAGACAAGGAGGGCTATTT** | +1 frameshift | **PQRETRRAI** | 1 (0.44) |
| **CCTCAAAGGAGAGACAAGAGGGCTATTT** | +1 frameshift | **PQRRDKRAI** | 1 (0.44) |
| **CCTCAAAGAAGAGACAAGAGGGCTATTT** | +1 frameshift | **PQRRDKRAI** | 1 (0.44) |
| **CCTCAAAGAGTAGACAAGAGGGCTATTT** | +1 frameshift | **PQRVDKRAI** | 2 (0.89) |
| **CCTCAAAGTAGAGACAAGAGGGCTATTT** | +1 frameshift | **PQSRDKRAI** | 1 (0.44) |
| **CCTCAAAGAGAGACGAAGAGGGCTGATTT** | +2 frameshift | **PQRETKRAD** | 1 (0.44) |
| **CCTCAAAGAGAGACCAAGAGGGCTATTTT** | +2 frameshift | **PQRETKRAI** | 1 (0.44) |
| **CCTCAAAAGAGAGACAAGAGGGGCTATTT** | +2 frameshift | **PQKRDKRGY** | 2 (0.89) |
| **CCTCAAAGAGAGACAAGGAGGGGCTATTT** | +2 frameshift | **PQRETRRGY** | 1 (0.44) |
| **CCTCAAAGGAGAGAACAAGAGGGCTATTT** | +2 frameshift | **PQRREQEGY** | 1 (0.44) |
| **CCTCAAAGGACGAGACAAGAGGGCTATTT** | +2 frameshift | **PQRTRQEGY** | 1 (0.44) |
| **CCTCAAAGGACGAGACAAGAGGGCTATTT** | +2 frameshift | **PQRTRQEGY** | 1 (0.44) |
| **CCTCAAAGAGAGACAAGAGGGCCTTATTT** | +2 frameshift | **PQRETRGPY** | 2 (0.89) |
| **CCTCAAACGAGAGACAAGAGGGCTTATTT** | +2 frameshift | **PQTRDKRAY** | 1 (0.44) |
| **CCTCAAACGAGAGACAAGAGGGGCTATTT** | +2 frameshift | **PQTRDKRGY** | 1 (0.44) |
| **CCTCGAAAGAGAGGACAAGAGGGCTATTT** | +2 frameshift | **PRKRGQEGY** | 1 (0.44) |
| **CCTCAAAGAGAGACGAAGATGGGCCTTATT** | insertion/substitution | **PQRETKMGLI**^†^ | 1 (0.44) |
| **CCTTCAAAAGAGAGACAAGAGGGGCTATTT** | insertion/substitution | **PSKERQEGLF**^†^ | 1 (0.44) |
| **CCTCAAAAGAGAGACAAGAGGGGCTCATTT** | insertion/substitution | **PQKRDKRGS**^†^ | 1 (0.44) |
| **CCTTCAAAGTAGACGTCACAAGAGGGCTATTT** | truncation/+2 frameshift | **PSK*TSQEGY** | 1 (0.44) |
|  |  | Total no. reads: | 225 |
|  |  | No. variants: | 35 [0.16] |

1. **H5N2 Passage 2**

| **HA_0_ cDNA nucleotide sequence^#^** | **Main mutational**  **effect** | **Translated**  **amino acids** | **No. of reads**  **(percentage)** |
| --- | --- | --- | --- |
| **CCTCAAAGAGAGACAAGAGGGCTATTT**^‡^ |  | **PQRETRGLF**^†^ | 33 (82.50) |
| **CCTCAAAGAGAGACAAGAGG_CTATTT** | +2 frameshift | **PQRETRGY** | 1 (2.50) |
| **CCTCAAAGAGAGACAAAGAGGGCTATTT** | +1 frameshift | **PQRETKRAI** | 1 (2.50) |
| **CCTCAAAGAGAGACAAGGAGGGCTATTT** | +1 frameshift | **PQRETRRAI** | 1 (2.50) |
| **CCTCAAAGAGAGACAAGACGGGCTACTT** | +1 frameshift | **PQRETRRAT** | 1 (2.50) |
| **CCTCAAAGGAGAGACAAGAGGGCTATTT** | +1 frameshift | **PQRRDKRAI** | 2 (5.00) |
| **CGCTCAAAGAGAGACAAGAGGGCTATTT** | +1 frameshift | **RSKRDKRAI** | 1 (2.50) |
|  |  | Total no. reads: | 40 |
|  |  | No. variants: | 7 [0.18] |

1. **H5N2 Passage 3**

| **HA_0_ cDNA nucleotide sequence^#^** | **Main mutational**  **effect** | **Translated**  **amino acids** | **No. of reads**  **(percentage)** |
| --- | --- | --- | --- |
| **CCTCAAAGAGAGACAAGAGGGCTATTT**^‡^ |  | **PQRETRGLF**^†^ | 46 (80.70) |
| **CCTGAAAGAGAGACAAGAGGGCTATTT** | substitution | **PERETRGLF**^†^ | 1 (1.75) |
| **CCTCAAAAAGAGACAAGGGGGCTATTT** | substitution | **PQKETRGLF**^†^ | 1 (1.75) |
| **CCTCAAAGAGAGACAAGGAGGGCTATTT** | +1 frameshift | **PQRETRRAI** | 1 (1.75) |
| **CCTCAAAGGAGAGACAAGAGGGCTATTT** | +1 frameshift | **PQRRDKRAI** | 2 (3.51) |
| **CCTCAAAGACGAGACAAGAGGGCTATTT** | +1 frameshift | **PQRRDKRAI** | 1 (1.75) |
| **CCTCAAAAGAGAGACAAAGAGGGCTATTT** | +2 frameshift | **PQKRDKEGY** | 1 (1.75) |
| **CCTCAAAGAGAGACAAGAGGGCTGAATTT** | +2 frameshift | **PQRETRGLN** | 1 (1.75) |
| **CCTCCCAAAGAGAGACAAGAGGGCTATTT** | +2 frameshift | **PPKERQEGY** | 1 (1.75) |
| **CCGTCAAAGGAGAGACAAGAGGGCTATTT** | +2 frameshift | **PSKERQEGY** | 2 (3.51) |
|  |  | Total no. reads: | 57 |
|  |  | No. variants: | 10 [0.18] |

1. **H5N2 Passage 4**

| **HA_0_ cDNA nucleotide sequence^#^** | **Main mutational**  **effect** | **Translated**  **amino acids** | **No. of reads**  **(percentage)** |
| --- | --- | --- | --- |
| **CCTCAAAGAGAGACAAGAGGGCTATTT**^‡^ |  | **PQRETRGLF**^†^ | 240 (92.66) |
| **CCT_AAAGAGAGACAAGAGGGCTATTT** | +2 frameshift | **PKERQEGY** | 1 (0.39) |
| **CCTCAAAGAGAGGCAAGAGGGCTATTT** | substitution | **PQREARGLF**^†^ | 2 (0.77) |
| **CCTCAAAGAGAGACAAGAGGGCGGTTT** | substitution | **PQRETRGRF**^†^ | 1 (0.39) |
| **CCTCAAAGAGAGACAAAAGGGCTATTT** | substitution | **PQRETKGLF**^†^ | 1 (0.39) |
| **CCTCAAAGAGAGACCAAGAGGGCTATTT** | +1 frameshift | **PQRETKRAI** | 1 (0.39) |
| **CCTCAAAGAGAGACAATGAGGGCTATTT** | +1 frameshift | **PQRETMRAI** | 1 (0.39) |
| **CCTCAAAGAGAGACAAGAGGGGCTATTT** | +1 frameshift | **PQRETRGAI** | 2 (0.77) |
| **CCTCAAAGAAGAGACAAGAGGGCTATTT** | +1 frameshift | **PQRRDKRAI** | 1 (0.39) |
| **CCTCAAAGGAGAGACAAGAGGGCTATTT** | +1 frameshift | **PQRRDKRAI** | 1 (0.39) |
| **CCTCGAAAGAGAGACAAGAGGGCTATTT** | +1 frameshift | **PRKRDKRAI** | 1 (0.39) |
| **CCTCGAACGAGAGACAAGAGGGCTATTT** | +1 frameshift | **PRTRDKRAI** | 1 (0.39) |
| **CCTCAAAGAGAGACAAGAAGGGACTATTT** | +2 frameshift | **PQRETRRDY** | 1 (0.39) |
| **CCTCAAAGAGAGATCAAGGAGGGCTATTT** | +2 frameshift | **PQREIKEGY** | 1 (0.39) |
| **CCTCAAAGGAGAGACAAGAGGGCTAGATTT** | insertion/substitution | **PQRRDKRARF**^†^ | 1 (0.39) |
| **CCTCAAACGAGAGCACCAAGAGGGCTATTT** | insertion/substitution | **PQTRAPRGLF**^†^ | 1 (0.39) |
| **CCTCGAAAGAAGAGACAAGAGGGCTCATTT** | insertion/substitution | **PRKKRQEGS**^†^ | 1 (0.39) |
| **CCTCGAAAGGACGACGACAATGAGGGGCTATTT** | insertion/substitution | **PRKDDDNEGLF**^†^ | 1 (0.39) |
|  |  | Total no. reads: | 259 |
|  |  | No. variants: | 18 [0.07] |

1. **H5N2 Passage 5**

| **HA_0_ cDNA nucleotide sequence^#^** | **Main mutational**  **effect** | **Translated**  **amino acids** | **No. of reads**  **(percentage)** |
| --- | --- | --- | --- |
| **CCTCAAAGAGAGACAAGAGGGCTATTT**^‡^ |  | **PQRETRGLF**^†^ | 267 (92.07) |
| **CCTCAAAGAGAGACAAGAGG_CTATTT** | +2 frameshift | **PQRETRGY** | 1 (0.35) |
| **CCTCAAAGAGAGACAAGAGGGCTATCC** | substitution | **PQRETRGLS**^†^ | 1 (0.35) |
| **CCTCGAAAGAGAGACAAGAGGGCTATTT** | +1 frameshift | **PRKRDKRAI** | 1 (0.35) |
| **CCTCAAAAGAGAGACAAGAGGGCTATTT** | +1 frameshift | **PQKRDKRAI** | 1 (0.35) |
| **CCTCAAAGAGAGGACAAGAGGGCTATTT** | +1 frameshift | **PQREDKRAI** | 1 (0.35) |
| **CCTCAAAGAGAGATCAAGAGGGCTATTT** | +1 frameshift | **PQREIKRAI** | 1 (0.35) |
| **CCTCAAAGAGAGACAAGAGGGCTTATTT** | +1 frameshift | **PQRETRGLI** | 2 (0.69) |
| **CCTCAAAGAGAGACAAGAGGGCTGATTT** | +1 frameshift | **PQRETRGLI** | 1 (0.35) |
| **CCTCAAAGAGAGACAAGAAGGGCTATTT** | +1 frameshift | **PQRETRRAI** | 1 (0.35) |
| **CCTCAAAGAGAGACAAGGAGGGCTATTT** | +1 frameshift | **PQRETRRAI** | 1 (0.35) |
| **CCTCAAAGAAGAGACAAGAGGGCTATTT** | +1 frameshift | **PQRRDKRAI** | 1 (0.35) |
| **CCTCAAAGAGTAGACAAGAGGGCTATTT** | +1 frameshift | **PQRVDKRAI** | 1 (0.35) |
| **CCTCGAA_GAGAGACAAGAGGGCTATTT** | substitution | **PRRETRGLF**^†^ | 2 (0.69) |
| **CCTACAAAGAGAGACAAGAGGGCTATTT** | +1 frameshift | **PTKRDKRAI** | 1 (0.35) |
| **CCTCAAAGAGAGACAAGAAGGGCTAATTT** | +2 frameshift | **PQRETRRAN** | 1 (0.35) |
| **CCTCAAACGAGAGACAAGGAGGGCTATTT** | +2 frameshift | **PQTRDKEGY** | 1 (0.35) |
| **CCTCGAAAGAGAGACCAAGAAGGGCTATTT** | insertion/substitution | **PRKRDQEGLF**^†^ | 1 (0.35) |
| **CCTCAAAAGAGAGACAAAGAGGGGCTTATTT** | insertion/substitution | **PQKRDKEGLI**^†^ | 1 (0.35) |
| **CCTCAAAGAGGTAGGACAAGAGGGGCTATTT** | +1 frameshift | **PQRGRTRGAI** | 1 (0.35) |
| **CCTCAAAGAGGTAGGACAAGAGGGGCTTATTT** | +1 frameshift | **PQRGRTRGAY** | 1 (0.35) |
| **CCTCAAAGAGAGACAAGAACCGGCACGGG__AGTTT** | +1 frameshift | **PQRETRTGTGV** | 1 (0.35) |
|  |  | Total no. reads: | 290 |
|  |  | No. variants: | 22 [0.08] |

1. **H5N2 Passage 6**

| **HA_0_ cDNA nucleotide sequence^#^** | **Main mutational**  **effect** | **Translated**  **amino acids** | **No. of reads**  **(percentage)** |
| --- | --- | --- | --- |
| **CCTCAAAGAGAGACAAGAGGGCTATTT**^‡^ |  | **PQRETRGLF**^†^ | 404 (95.06) |
| **CCTCAAAGAGAGACAAGAGG_CTATTT** | +2 frameshift | **PQRETRGY** | 1 (0.24) |
| **CCTCCAAGAGAGACAAGAGGGCTATTT** | substitution | **PPRETRGLF**^†^ | 1 (0.24) |
| **CCTCAAAAAGAGACAAGAGGGCTATTT** | substitution | **PQKETRGLF**^†^ | 1 (0.24) |
| **CCTCAAAGAGAGACAAGAGGGCTAGTA** | substitution | **PQRETRGLV**^†^ | 1 (0.24) |
| **CCTCAAAGAGAGACAAGAGGGCAATTT** | substitution | **PQRETRGQF**^†^ | 2 (0.47) |
| **CCTCCAAAGAGAGACAAGAGGGCTATTT** | +1 frameshift | **PPKRDKRAI** | 1 (0.24) |
| **CCTCAAAGAGAGACAAGGAGGGCTATTT** | +1 frameshift | **PQRETRRAI** | 3 (0.71) |
| **CCTCAA_GAGAGACAACGAGGGCTATTT** | substitution | **PQERQRGLF**^†^ | 2 (0.47) |
| **CCTCAAAGGAGAGACAAGAGGGCTATTT** | +1 frameshift | **PQRRDKRAI** | 1 (0.24) |
| **CCTCAAAGAGAGACAAGAGGGGCTATTT** | +1 frameshift | **PQRETRGAI** | 1 (0.24) |
| **CCTCAAAGTAGAGACAAGAGGGCTATTT** | +1 frameshift | **PQSRDKRAI** | 1 (0.24) |
| **CCTCAAACGAGAGACAAGAGGGCTATTT** | +1 frameshift | **PQTRDKRAI** | 2 (0.47) |
| **CCTCAAAGAGAGATCAAGAGGGCTTATTT** | +2 frameshift | **PQREIKRAY** | 1 (0.24) |
| **CCTCAAAGAGAGACAAAGAGGGGCTATTT** | +2 frameshift | **PQRETKRGY** | 1 (0.24) |
| **CCTCAAAGAAGAGGACAAGAGGGCTATTA** | +2 frameshift | **PQRRGQEGY** | 1 (0.24) |
| **CCTCAAAGAGAGACAAGAACCGGCACGGG__AGTTT** | +1 frameshift | **PQRETRTGTGV** | 1 (0.24) |
|  |  | Total no. reads: | 425 |
|  |  | No. variants: | 17 [0.04] |

1. **H5N2 Passage 7**

| **HA_0_ cDNA nucleotide sequence^#^** | **Main mutational**  **effect** | **Translated**  **amino acids** | **No. of reads**  **(percentage)** |
| --- | --- | --- | --- |
| **CCTCAAAGAGAGACAAGAGGGCTATTT**^‡^ |  | **PQRETRGLF**^†^ | 82 (71.93) |
| **CCTCAA_GAGAGACA_GAGGGCTATTT** | +1 frameshift | **PQERQRAI** | 2 (1.75) |
| **CCT_AA_GAGAGACAAGAGGGCTATTT** | +1 frameshift | **PKRDKRAI** | 1 (0.88) |
| **CCTCAA_GAGAGGACAAGAGGGCTATTT** | substitution | **PQERTRGLF**^†^ | 1 (0.88) |
| **CCTCGAA_GAGAGACAAGAGGGCTATTT** | substitution | **PRRETRGLF**^†^ | 1 (0.88) |
| **CCTCCAAAGAGAGACAAGAGGGCTATTT** | +1 frameshift | **PPKRDKRAI** | 1 (0.88) |
| **CCTCAAAAGAGAGACAAGAGGGCTATTT** | +1 frameshift | **PQKRDKRAI** | 1 (0.88) |
| **CCTCAAAGAGAGATCAAGAGGGCTATTT** | +1 frameshift | **PQREIKRAI** | 1 (0.88) |
| **CCTCAAAGAGAGACCAAGAGGGCTATTT** | +1 frameshift | **PQRETKRAI** | 1 (0.88) |
| **CCTCAAAGAGAGACAAGAGGGGCTATTT** | +1 frameshift | **PQRETRGAI** | 1 (0.88) |
| **CCTCAAAGAGAGACAAGAAGGGCTATTT** | +1 frameshift | **PQRETRRAI** | 1 (0.88) |
| **CCTCAAAGAGAGACAAGATGGGCTATTT** | +1 frameshift | **PQRETRWAI** | 1 (0.88) |
| **CCTCAAAGAGAGACAACGAGGGCTATTT** | +1 frameshift | **PQRETTRAI** | 1 (0.88) |
| **CCTCAAAGAAGAGACAAGAGGGCTATTT** | +1 frameshift | **PQRRDKRAI** | 1 (0.88) |
| **CCTCAAACGAGAGACAAGAGGGCTATTT** | +1 frameshift | **PQTRDKRAI** | 2 (1.75) |
| **CCTTCAAAGAGAGACAAGAGGGCTATTT** | +1 frameshift | **PSKRDKRAI** | 1 (0.88) |
| **CCTCGAA_GAGAGGACAAGAGGGCTATTT** | +1 frameshift | **PRREDKRAI** | 1 (0.88) |
| **CCTCAAAGAGAGGACAAGAGGGCTTATTT** | +2 frameshift | **PQREDKRAY** | 1 (0.88) |
| **CCTCAAAGAGAGATCAAGAGGGGCTATTT** | +2 frameshift | **PQREIKRGY** | 1 (0.88) |
| **CCTCAAAGAGAGACCAAGACGGGCTATTT** | +2 frameshift | **PQRETKTGY** | 1 (0.88) |
| **CCTCAAAGAGGAGACAAGAGGGCTAGTTT** | +2 frameshift | **PQRGDKRAS** | 1 (0.88) |
| **CCTCAAAGAAGAGGACAAGAGGGCTATTT** | +2 frameshift | **PQRRGQEGY** | 1 (0.88) |
| **CCTCAAACGAGACGACAAGAGGGCTATTT** | +2 frameshift | **PQTRRQEGY** | 1 (0.88) |
| **CCTACAAAGAGAGACAAGAGGGCTAGTTT** | +2 frameshift | **PTKRDKRAS** | 1 (0.88) |
| **CCTCAAAGAGAGACAAGAGGGGCCTATTT** | +2 frameshift | **PQRETRGAY** | 1 (0.88) |
| **CCTACAAAGAGAGACCAAGAGGGCTATTT** | +2 frameshift | **PTKRDQEGY** | 1 (0.88) |
| **CCTCCAAAGAGAGTACAAGAGGGCTATTT** | +2 frameshift | **PPKRVQEGY** | 1 (0.88) |
| **CCTCAAAGAGAGACAAGAGGGCTAGGTTT** | +2 frameshift | **PQRETRGLG** | 1 (0.88) |
| **CCTCCAAAGAGACGACAACGAGGGCTATTT** | insertion/substitution | **PPKRRQRGLF**^†^ | 1 (0.88) |
| **CCTCGAAAGAGAGTACCAATGAGGGCTATTT** | +1 frameshift | **PRKRVPMRAI** | 1 (0.88) |
|  |  | Total no. reads: | 114 |
|  |  | No. variants: | 30 [0.26] |

1. **H5N2 Passage 11**

| **HA_0_ cDNA nucleotide sequence^#^** | **Main mutational**  **effect** | **Translated**  **amino acids** | **No. of reads**  **(percentage)** |
| --- | --- | --- | --- |
| **CCTCAAAGAGAGACAAGGGGGCTATTT**^‡^ |  | **PQRETRGLF**^†^ | 134 (64.42) |
| **CCT_AAAGAGAGACAAGAGGGCTATTT** | +2 frameshift | **PKERQEGY** | 1 (0.48) |
| **CCTCAAAGAGAGACAAGAGG_CTATTT** | +2 frameshift | **PQRETRGY** | 9 (4.33) |
| **CCTCAAAGAGGGACAAGAGGGCTATTT** | substitution | **PQRGTRGLF**^†^ | 1 (0.48) |
| **CCTCAAGAGAGACGAAGAGGGCTATTT** | substitution | **PQERRRGLF**^†^ | 1 (0.48) |
| **CCTCAAAGAGAAGACA_GAGGGCTATTT** | substitution | **PQREDRGLF**^†^ | 1 (0.48) |
| **CCTCAAAGAGAAGACA_GAGGGCTATTT** | substitution | **PQREDRGLF**^†^ | 1 (0.48) |
| **CCTCCAAAGAGAGACAAGAGG_CTATTT** | substitution | **PPKRDKRLF**^†^ | 1 (0.48) |
| **CCTCAAAGAGAGACCAAGAGG_CTATTT** | substitution | **PQRETKRLF**^†^ | 1 (0.48) |
| **CCTCAAAGAGAGACAATGAGG_CTATTT** | substitution | **PQRETMRLF**^†^ | 1 (0.48) |
| **CCTCAAAGAGAGACAAGAGGGCTAATTT** | +1 frameshift | **PQRETRGLI** | 2 (0.96) |
| **CCTCAAAGAGAGACAAGAGGGCTTATTT** | +1 frameshift | **PQRETRGLI** | 1 (0.48) |
| **CCTCAAAGAGAGACAAGAGGGCCTATTT** | +1 frameshift | **PQRETRGPI** | 1 (0.48) |
| **CCTCTAAAGAGAGACAAGAGGGCTATTT** | +1 frameshift | **PLKRDKRAI** | 1 (0.48) |
| **CCTCCAAAGAGAGACAAGAGGGCTATTT** | +1 frameshift | **PPKRDKRAI** | 3 (1.44) |
| **CCTCAAAGAGAGGACAAGAAGGCTATTT** | +1 frameshift | **PQREDKKAI** | 1 (0.48) |
| **CCTCAAAGAGAGGACAAGAGGGCTATTT** | +1 frameshift | **PQREDKRAI** | 1 (0.48) |
| **CCTCAAAAGAGAGACAAGAGGGCTATTT** | +1 frameshift | **PQKRDKRAI** | 1 (0.48) |
| **CCTCAAAGAGATGACAAGAGGGCTATTT** | +1 frameshift | **PQRDDKRAI** | 1 (0.48) |
| **CCTCAA_GAGAGACGAATGAGGGCTATTT** | +1 frameshift | **PQERRMRAI** | 1 (0.48) |
| **CCTCAAAGAGAGACAAAGAGGGCTATTT** | +1 frameshift | **PQRETKRAI** | 5 (2.40) |
| **CCTCAAAGAGAGACAAGGAGGGCTATTT** | +1 frameshift | **PQRETRRAI** | 1 (0.48) |
| **CCTCAAAGGAGAGACAAGAGGGCTATTT** | +1 frameshift | **PQRRDKRAI** | 3 (1.44) |
| **CCTCAAAGAAGAGACAAGAGGGCTATTT** | +1 frameshift | **PQRRDKRAI** | 2 (0.96) |
| **CCTCAAAGTAGAGACAAGAGGGCTATTT** | +1 frameshift | **PQSRDKRAI** | 1 (0.48) |
| **CCTCAAACGAGAGACAAGAGGGCTATTT** | +1 frameshift | **PQTRDKRAI** | 1 (0.48) |
| **CCTCGAAAGAGAGACAAGAGGGCTATTT** | +1 frameshift | **PRKRDKRAI** | 1 (0.48) |
| **CCTCAAAGAGAGACAACGAGGGCTATTT** | +1 frameshift | **PQRETTRAI** | 1 (0.48) |
| **CCTCAAAGAGAGACCAAGGAGG_CTATTT** | +1 frameshift | **PQRETKEAI** | 1 (0.48) |
| **CCTCAAAGAGAGATCAAGAGGGCTTATTT** | +2 frameshift | **PQREIKRAY** | 3 (1.44) |
| **CCTCAAAGAGAGACGAAGAGGGCTGATTT** | +2 frameshift | **PQRETKRAD** | 1 (0.48) |
| **CCTCAAAGAGACGACAAGAGGGGCTATTT** | +2 frameshift | **PQRDDKRGY** | 1 (0.48) |
| **CCTCAAAGAGAAGACAAAGGAGGCTATTT** | +2 frameshift | **PQREDKGGY** | 1 (0.48) |
| **CCTCAAAGAGAGGACAAGAGGGCTGATTT** | +2 frameshift | **PQREDKRAD** | 1 (0.48) |
| **CCTCAAAGAGAGGACAAGAGGGCTTATTT** | +2 frameshift | **PQREDKRAY** | 2 (0.96) |
| **CCTCAAAGAGAGATCAAAGAGGGCTATTT** | +2 frameshift | **PQREIKEGY** | 1 (0.48) |
| **CCTCAAAGAGAGACAAGAAGGGCTGATTT** | +2 frameshift | **PQRETRRAD** | 1 (0.48) |
| **CCTCAAAGAAGAGACGAAGAGG_CTATTT** | +2 frameshift | **PQRRDEEAI** | 1 (0.48) |
| **CCTCAAAGAGAGACAAGGAGGGCTACTTT** | +2 frameshift | **PQRETRRAT** | 1 (0.48) |
| **CCTCAAAGAGAGACAAGAAGGGGCTATTT** | +2 frameshift | **PQRETRRGY** | 1 (0.48) |
| **CCTCAAAGGAGAGACAAGAGGGGCTATTT** | +2 frameshift | **PQRRDKRGY** | 1 (0.48) |
| **CCTCAAAGGAGAGATCAAGAGGGCTATTT** | +2 frameshift | **PQRRDQEGY** | 1 (0.48) |
| **CCTCAAAGACGACGACAAGAGGGCTATTT** | +2 frameshift | **PQRRRQEGY** | 1 (0.48) |
| **CCTCAAAGAAGAGGACAAGAGGGCTATTT** | +2 frameshift | **PQRRGQEGY** | 1 (0.48) |
| **CCTTCCAAAGAGAGACAAGAGGGCTATTT** | +2 frameshift | **PSKERQEGY** | 1 (0.48) |
| **CCGTCAAAGGAGAGACAAGAGGGCTATTT** | +2 frameshift | **PSKERQEGY** | 1 (0.48) |
| **CCTCAA_GAAGAGCACAAGAGGGCTTATTT** | +2 frameshift | **PQEEHKRAY** | 1 (0.48) |
| **CCTCAAAGAAGAGGACAAGAGGGCTTATTT** | insertion/substitution | **PQRRGQEGLF**^†^ | 3 (1.44) |
| **CCTCAAAAGCAGATGACAAGAGGGCTAATTT** | insertion/substitution | **PQKQMTRGLI**^†^ | 1 (0.48) |
| **CCTCAAAGGAGAAGGAACAAGAGGGCTATTT** | +1 frameshift | **PQRRRNKRAI** | 1 (0.48) |
| **CCTCAAAGACGAGACCAACGAGGGCTAGATTT** | insertion/substitution | **PQRRDQRGLD**^†^ | 1 (0.48) |
|  |  | Total no. reads: | 208 |
|  |  | No. variants: | 51 [0.25] |

1. **H5N2 Passage 15**

| **HA_0_ cDNA nucleotide sequence^#^** | **Main mutational**  **effect** | **Translated**  **amino acids** | **No. of reads**  **(percentage)** |
| --- | --- | --- | --- |
| **CCTCAAAGAGAGACAAGAGGGCTATTT**^‡^ |  | **PQRETRGLF**^†^ | 146 (47.71) |
| **CCT_AAAGAGAGACAAGAGG__TATTT** | codon deletion | **PKERQEVF**^†^ | 1 (0.33) |
| **CCTCAA_GAGAGACA_GAGGGCTATTT** | +1 frameshift | **PQERQRAI** | 1 (0.33) |
| **CCTCAAAGAGAGACAA__CCAGAGTTT** | +1 frameshift | **PQRETTRV** | 1 (0.33) |
| **CCTCAAAG_GAGACAAGAGGGCTATTT** | +2 frameshift | **PQRRQEGY** | 1 (0.33) |
| **CCTCAA_GAGAGACA_GAGGGCTAGTTT** | +2 frameshift | **PQERQRAS** | 1 (0.33) |
| **CCT_AAAGAGAGACAAGAGGGCTATTT** | +2 frameshift | **PKERQEGY** | 1 (0.33) |
| **CCTAACAAGGAGACAAGAGGGCTATTT** | substitution | **PNKETRGLF**^†^ | 1 (0.33) |
| **CCTCAAAGAGAGGCAAGAGGGCTATTT** | substitution | **PQREARGLF**^†^ | 3 (1.00) |
| **CCTCAA_GGAGAGAACAAGAGG_CTATTT** | substitution | **PQGENKRLF**^†^ | 1 (0.33) |
| **CCTCAAAAAGAGACAAGAGGGCTATTT** | substitution | **PQKETRGLF**^†^ | 2 (0.66) |
| **CCTCAAAGAGAGAGAAGAGGGCTATTT** | substitution | **PQRERRGLF**^†^ | 3 (1.00) |
| **CCTCAAAGAGAGACAAGAGGGCGATTT** | substitution | **PQRETRGRF**^†^ | 1 (0.33) |
| **CCTCAAAGAGGGACAAGAGGGCTATTT** | substitution | **PQRGTRGLF**^†^ | 3 (1.00) |
| **CCTCAAAGAAAGACAAGAGGGCTATTT** | substitution | **PQRKTRGLF**^†^ | 1 (0.33) |
| **CCTCAA_GAGAGACAACGAGGGCTATTT** | substitution | **PQERQRGLF**^†^ | 1 (0.77) |
| **CCTCAA_GAGAGACGAAGAGGGCTATTT** | substitution | **PQERRRGLF**^†^ | 1 (0.77) |
| **CCTCAAAAGAGAGACAAGAGGGCTATTT** | +1 frameshift | **PQKRDKRAI** | 2 (0.66) |
| **CCTCAAAGAGATGACAAGAGGGCTATTT** | +1 frameshift | **PQRDDKRAI** | 4 (1.33) |
| **CCTCCAAAGAGAGACAAGAGGGCTATTT** | +1 frameshift | **PPKRDKRAI** | 7 (2.32) |
| **CCTCCAAAGAGAGACAAGAGGGCTAGTT** | +1 frameshift | **PPKRDKRAS** | 1 (0.33) |
| **CCTCAAAGAGAAGACAAGAGGGCTATTT** | +1 frameshift | **PQREDKRAI** | 1 (0.33) |
| **CCTCAAAGAGAGGACAAGAGGGCTATTT** | +1 frameshift | **PQREDKRAI** | 4 (1.33) |
| **CCTCAAAGAGAGCACAAGAGGGCTATTT** | +1 frameshift | **PQREHKRAI** | 4 (1.33) |
| **CCTCAAAGAGAGATCAAGAGGGCTATTT** | +1 frameshift | **PQREIKRAI** | 2 (0.66) |
| **CCTCAAAGAGAGACAGAGAGGGCTATTT** | +1 frameshift | **PQRETERAI** | 2 (0.66) |
| **CCTCAAAGAGAGACCAAGAGGGCTATTT** | +1 frameshift | **PQRETKRAI** | 10 (3.32) |
| **CCTCAAAGAGAGACAATGAGGGCTATTT** | +1 frameshift | **PQRETMRAI** | 2 (0.66) |
| **CCTCAAAGAGAGACAAGAGGGGCTATTT** | +1 frameshift | **PQRETRGAI** | 28 (9.30) |
| **CCTCAAAGAGAGACAAGAGGGCTAATTT** | +1 frameshift | **PQRETRGLI** | 5 (1.66) |
| **CCTCAAAGAGAGACAAGAGGGCTCATTT** | +1 frameshift | **PQRETRGLI** | 3 (1.00) |
| **CCTCAAAGAGAGACAAGAAGGGCTATTT** | +1 frameshift | **PQRETRRAI** | 10 (3.32) |
| **CCTCAAAGAGAGACAAGATGGGCTATTT** | +1 frameshift | **PQRETRWAI** | 1 (0.33) |
| **CCTCAAAGAGAGACAACGAGGGCTATTT** | +1 frameshift | **PQRETTRAI** | 2 (0.66) |
| **CCTCAAAGAGAGTACAAGAGGGCTATTT** | +1 frameshift | **PQREYKRAI** | 2 (0.66) |
| **CCTCAAAGAGGAGACAAGAGGGCTATTT** | +1 frameshift | **PQRGDKRAI** | 1 (0.33) |
| **CCATCAAAGAGAGACAAGAGGGCTATTT** | +1 frameshift | **PSKRDKRAI** | 1 (0.33) |
| **CCTCAAAGGAGAGACAAGAGGGCTATTT** | +1 frameshift | **PQRRDKRAI** | 3 (1.00) |
| **CCTCAAAGAAGAGACAAGAGGGCTATTT** | +1 frameshift | **PQRRDKRAI** | 1 (0.33) |
| **CCTACAAAGAGAGACAAGAGGGCTATTT** | +1 frameshift | **PTKRDKRAI** | 3 (1.00) |
| **CCTCAAACGAGAGACAAGAGGGCTATTT** | +1 frameshift | **PQTRDKRAI** | 1 (0.33) |
| **CCTCGAAAGAGAGACAAGAGGGCTATTT** | +1 frameshift | **PRKRDKRAI** | 1 (0.33) |
| **CCTCAA_GAGAGATCAAGGAGGGCTATTT** | +1 frameshift | **PQERSRRAI** | 1 (0.33) |
| **CCTCAAAGAGAGACCAAGGAGGGCTATTT** | +2 frameshift | **PQRETKEGY** | 1 (0.33) |
| **CCTCAAAGAGAGACGAAGAGGGCTACTTT** | +2 frameshift | **PQRETKRAT** | 2 (0.66) |
| **CCTCAAAGAGAGACCAAGAGGGCTTATTT** | +2 frameshift | **PQRETKRAY** | 1 (0.33) |
| **CCTCAAAGAGAGACCAAGAGGGGCTATTT** | +2 frameshift | **PQRETKRGY** | 1 (0.33) |
| **CCTCAAAGAGAGACAACGAGGGCTCATTT** | +2 frameshift | **PQRETTRAH** | 1 (0.33) |
| **CCTCAAAAGAGAGACAAGAGGGGCTATTT** | +2 frameshift | **PQKRDKRGY** | 1 (0.33) |
| **CCTCAAAAGAGAGACGAAGAGGGCTATTT** | +2 frameshift | **PQKRDEEGY** | 1 (0.33) |
| **CCTCAAAGAGAGACAACGAGGGCTAGTTT** | +2 frameshift | **PQRETTRAS** | 1 (0.33) |
| **CCTCAAAGACGAGACAAGAGGGGCTATTT** | +2 frameshift | **PQRRDKRGY** | 1 (0.33) |
| **CCTCAAAGAAGAGACCAAGAGGGCTATTT** | +2 frameshift | **PQRRDQEGY** | 1 (0.33) |
| **CCTCAAAGAAGAGGACAAGAGG_CTTATT** | +2 frameshift | **PQRRGQEAY** | 1 (0.33) |
| **CCTCGAAAGAGAGACAAGAGGGCTAGTTT** | +2 frameshift | **PRKRDKRAS** | 1 (0.33) |
| **CCTCGAAAGAGAGACAAGAGGGGCTATTT** | +2 frameshift | **PRKRDKRGY** | 1 (0.33) |
| **CCTTCAAAGAGAGACAAGAGGGCTCATTT** | +2 frameshift | **PSKRDKRAH** | 1 (0.33) |
| **CCTCAAGTAGATGACAAGAGGGCTACTTT** | +2 frameshift | **PQVDDKRAT** | 2 (0.66) |
| **CCTCGAAAGAGAGACAAGAGGGCTCATTT** | +2 frameshift | **PRKRDKRAH** | 1 (0.33) |
| **CCGTCAAAGAGAGACAAGAGGGGCTATTT** | +2 frameshift | **PSKRDKRGY** | 1 (0.33) |
| **CCTTCAAAGAGAGACAACGAGGGCTATTT** | +2 frameshift | **PSKRDNEGY** | 1 (0.33) |
| **CCTCAAAGAGAGATAAGATGGGGCTATTT** | +2 frameshift | **PQREIRWGY** | 1 (0.33) |
| **CCTCCAAAGAGAGACAAGAGGGCTCGATTT** | insertion/substitution | **PPKRDKRARF**^†^ | 1 (0.33) |
| **CCTCAAAAGAAGAGACAAGACGGGCTATTT** | insertion/substitution | **PQKKRQDGLF**^†^ | 1 (0.33) |
| **CCTCAAAGAGATGACAAGGAGGGCTACTTT** | insertion/substitution | **PQRDDKEGYF**^†^ | 1 (0.33) |
| **CCTCAAAGAAGAGACGAAGAAGGGCTATTT** | insertion/substitution | **PQRRDEEGLF**^†^ | 1 (0.33) |
| **CCTCAAAGAAGAGGACAAGAGGGCTTATTT** | insertion/substitution | **PQRRGQEGLF**^†^ | 1 (0.33) |
| **CCTCAAAGACGTAGACAAGAGGGCTTATTT** | insertion/substitution | **PQRRRQEGLF**^†^ | 1 (0.33) |
| **CCTCCAAAGAGACGACAAGAGGGCTCGACTTT** | +2 frameshift | **PPKRRQEGST** | 2 (0.66) |
| **CCTCAAAGAGATGCACCAAAGGAGGGCTATTT** | +2 frameshift | **PQRDAPKEGY** | 1 (0.33) |
|  |  | Total no. reads: | 301 |
|  |  | No. variants: | 70 [0.23] |

1. **H5N2 Passage 17**

| **HA_0_ cDNA nucleotide sequence^#^** | **Main mutational**  **effect** | **Translated**  **amino acids** | **No. of reads**  **(percentage)** |
| --- | --- | --- | --- |
| **CCTCAAAGAGAGACAAGAGGGCTATTT**^‡^ |  | **PQRETRGLF**^†^ | 81 (62.31) |
| **CCTCAA_GAGAGACA_GAGGGCTATTT** | +1 frameshift | **PQERQRAI** | 3 (2.31) |
| **CCTCAAAGAGAGACAAGAGG_CTATTT** | +2 frameshift | **PQRETRGY** | 1 (0.77) |
| **CCTCAAAGAGAAGACAAGAGGCTATTT** | substitution | **PQREDKRLF**^†^ | 1 (0.77) |
| **CCTCAAAGAGAGATAAGAGGGCTATTT** | substitution | **PQREIRGLF**^†^ | 1 (0.77) |
| **CCTCAAAGAGAGACAAGAGGGCTGATT** | substitution | **PQRETRGLI**^†^ | 1 (0.77) |
| **CCTCAAAGAGAGACAAGAGG_CTCATTT** | substitution | **PQRETRGSF**^†^ | 1 (0.77) |
| **CCTCAA_GAGAGAGCAAGAGGGCTATTT** | substitution | **PQERARGLF**^†^ | 1 (0.77) |
| **CCTCAAAGAGAGATCAAGAGG_CTATTT** | substitution | **PQREIKRLF**^†^ | 1 (0.77) |
| **CCTCCAAAGAGAGACAAGAGGGCTATTT** | +1 frameshift | **PPKRDKRAI** | 1 (0.77) |
| **CCTCAAAGAGATGACAAGAGGGCTATTT** | +1 frameshift | **PQRDDKRAI** | 2 (1.54) |
| **CCTCGAAAGAGAGACAAGAGGGCTATTT** | +1 frameshift | **PRKRDKRAI** | 2 (1.54) |
| **CCTCAAAGAGTAGACAAGAGGGCTATTT** | +1 frameshift | **PQRVDKRAI** | 1 (0.77) |
| **CCTCAAAGAGAGTACAAGAGGGCTATTT** | +1 frameshift | **PQREYKRAI** | 1 (0.77) |
| **CCTCAAAGAAGAGACAAGAGGGCTATTT** | +1 frameshift | **PQRRDKRAI** | 2 (1.54) |
| **CCTCAAAGAGAGCACAAGAGGGCTATTT** | +1 frameshift | **PQREHKRAI** | 1 (0.77) |
| **CCTCAAAGAGAGACGAAGAGGGCTATTT** | +1 frameshift | **PQRETKRAI** | 1 (0.77) |
| **CCTCAAAGAGAGACAAGAGGGCTTATTT** | +1 frameshift | **PQRETRGLI** | 2 (1.54) |
| **CCTCAAAGAGAGACAAGAGGGCTAATTT** | +1 frameshift | **PQRETRGLI** | 2 (1.54) |
| **CCTCAAAGAGAGACAAGAGGGCCTATTT** | +1 frameshift | **PQRETRGPI** | 1 (0.77) |
| **CCTCAAAGAGAGACAAGGAGGGCTATTT** | +1 frameshift | **PQRETRRAI** | 1 (0.77) |
| **CCTCAA_GTAGATGACAAGAGGGCTACTTC** | +1 frameshift | **PQVDDKRAT** | 1 (0.77) |
| **CCTCAAAGAGAGCACAAGAGGGCTACTTT** | +2 frameshift | **PQREHKRAT** | 1 (0.77) |
| **CCTCAAAGAGAGGACAAGAGGGGCTATTT** | +2 frameshift | **PQREDKRGY** | 1 (0.77) |
| **CCTCAAAGACGAGATCAAGAGGGCTTATTT** | insertion/substitution | **PQRRDQEGLF**^†^ | 1 (0.77) |
| **CCTCAAAGAAGAGGACAAGAGGGCTTATTT** | insertion/substitution | **PQRRGQEGLF**^†^ | 2 (1.54) |
| **CCTCAAAGAGTACGGACGAACGAGGGCCTATTT** | insertion/substitution | **PQRVRTNEGLF**^†^ | 1 (0.77) |
| **CCTCAAAGAGAGACAAGGAGGGCCTTATTT** | insertion/substitution | **PQRETRRALF**^†^ | 1 (0.77) |
| **CCTCAAAAGGAGATGACAACGAGGGCTATTT** | +1 frameshift | **PQKEMTTRAI** | 1 (0.77) |
| **CCTCAAAACGAGAGATCGAAGAGGGCTAGTT** | +1 frameshift | **PQNERSKRAS** | 1 (0.77) |
| **CCTCAAAGTACGACGATCAAGAGGGCTATTT** | +1 frameshift | **PQSTTIKRAI** | 1 (0.77) |
| **CCTCGAAAGAGTAGACGAACGAGGGCTATTT** | +1 frameshift | **PRKSRRTRAI** | 1 (0.77) |
| **CCTCAA_GAACGAGACAAGATGGGCCTATTT** | insertion/substitution | **PQERDKMGLF**^†^ | 1 (0.77) |
| **CCTCAAAGAGAGACAAGACGAGAAAGCAGGAATCCTGGCAATGCTGAAATTGAAGATCTCATCTTT** | insertion/substitution | **PQRETRRESRNPGNAEIEDLIF**^†^ | 1 (0.77) |
|  |  | Total no. reads: | 130 |
|  |  | No. variants: | 34 [0.26] |

^#^Insertions, deletions and substitutions in relation to the conventional low pathogenic sequence^‡^ are underlined; stop codons are indicated by “_*_”.

^†^In-frame HA_2_

[ ] total number of variants: total number of HACS read ratio
