# Supplementary material for: Emergence of highly pathogenic H5N2 and H7N1 influenza A viruses from low pathogenic precursors by serial passage in ovo
Source: PLoS One. 2020 Oct 8;15(10):e0240290. doi: 10.1371/journal.pone.0240290 (PMC7544131; doi:10.1371/journal.pone.0240290)
Supplement: S4 Table — #Insertions, deletions and substitutions in relation to the conventional low pathogenic sequence‡ are underlined; stop codons are indicated by “*”. †In-frame HA2. [] total number of variants: total number of HACS read ratio. (DOCX) [file pone.0240290.s004.docx]

**S4 Table: Variants detected at the hemagglutinin cleavage site (HA_0_) of H7N1 low pathogenic avian influenza viruses passaged in 14-day old embryonated chicken eggs**

1. **H7N1 Passage 1**

| **HA_0_ cDNA nucleotide sequence^#^** | **Main mutational**  **effect** | **Translated**  **amino acids** | **No. of reads**  **(percentage)** |
| --- | --- | --- | --- |
| **CCCGAACTCCCAAAGGGAAGAGGCCTGTTT**^‡^ |  | **PELPKGRGLF^†^** | 1690 (95.05) |
| **CCCGAACTCCCAAAGGGAA_G_GCCTGTTT** | +2 frameshift | **PELPKGRPV** | 6 (0.34) |
| **CCCGAACTCC_AA_GGGAAGAGGCCTGTTT** | +2 frameshift | **PELQGKRPV** | 1 (0.06) |
| **CCCGAA_TCCCAA_GGGAAGAGGCCTGTTT** | +2 frameshift | **PESQGKRPV** | 1 (0.06) |
| **CCC_AACTCC_AAAGGGAAGAGGCCTGTTT** | +2 frameshift | **PNSKGKRPV** | 1 (0.06) |
| **CC_GAACTCCCAA_GGGAAGAGGCCTGTTT** | +2 frameshift | **PNSQGKRPV** | 1 (0.06) |
| **CCCGAACTCTCAAAGGGAAGAGGCCTGTTT** | substitution | **PELSKGRGLF^†^** | 8 (0.45) |
| **CCCGAACTCCCAAAGGGAAGAGGCCTGCTT** | substitution | **PELPKGRGLL^†^** | 2 (0.11) |
| **CCCGAACTCCCAAAGGGAAGAGGCCTGTCT** | substitution | **PELPKGRGLS^†^** | 1 (0.06) |
| **CCCGAACTCCCAAGGGGAAGAGGCCTGTTT** | substitution | **PELPRGRGLF^†^** | 7 (0.39) |
| **CCCGAACTCCCAAAGGGAAGAGGCCCGTTT** | substitution | **PELPKGRGPF^†^** | 1 (0.06) |
| **CCCGACCTCCCAAAGGGAAGAGGCCTGTTT** | substitution | **PDLPKGRGLF^†^** | 1 (0.06) |
| **CCCGAACGCCCAAAGGGAAGAGGCCTGTTT** | substitution | **PERPKGRGLF^†^** | 1 (0.06) |
| **CC_GAACTCCCAA_GGGAAGAGGCCGTGTTT** | +1 frameshift | **PNSQGKRPC** | 1 (0.06) |
| **CC_GAACTCCCAA_GGGAAGAGGCCGTGTTT** | +1 frameshift | **PNSQGKRPC** | 1 (0.06) |
| **CCCGAACTCCCAAAGGGAAGAGGGCCTGTTT** | +1 frameshift | **PELPKGRGPV** | 5 (0.28) |
| **CCCGAAGCTCCCAAAGGGAAGAGGCCTGTTT** | +1 frameshift | **PEAPKGKRPV** | 1 (0.06) |
| **CCCCGAACTCCCAAAGGGAAGAGGCCTGTTT** | +1 frameshift | **PRTPKGKRPV** | 1 (0.06) |
| **CCCGAACTCCCAAAGGGGAAGAGGCCTGTTT** | +1 frameshift | **PELPKGKRPV** | 15 (0.84) |
| **CCCGAACTCCCAAAGGGAAGAGGCCATGTTT** | +1 frameshift | **PELPKGRGHV** | 1 (0.06) |
| **CCCGAACTCCCAAAGGGAAGAGGCCTGATTT** | +1 frameshift | **PELPKGRGLI** | 1 (0.06) |
| **CCCGAACTCCCAAAGGGAAGAGGCCGTGTTT** | +1 frameshift | **PELPKGRGRV** | 1 (0.06) |
| **CCCGAACTCCCAAAGGGAAGACGGCCTGTTT** | +1 frameshift | **PELPKGRRPV** | 5 (0.28) |
| **CCCGAACTCCCAAAGGGAAGAGGCCTTGTTT** | +1 frameshift | **PELPKGRGLV** | 7 (0.39) |
| **CCCGAACTCCCAAAGGGAAGTAGGCCTGTTT** | +1 frameshift | **PELPKGSRPV** | 1 (0.06) |
| **CCCGAACTCCCAAAGGGAACGAGGCCTGTTT** | +1 frameshift | **PELPKGTRPV** | 1 (0.06) |
| **CCCGAACTCCCAAACGGGAAGAGGCCTGTTT** | +1 frameshift | **PELPNGKRPV** | 1 (0.06) |
| **CCCGAAACTCCCAAAGGGAAGAGGCCTGTTT** | +1 frameshift | **PETPKGKRPV** | 2 (0.11) |
| **CCCGGAACTCCCAAAGGGAAGAGGCCTGTTT** | +1 frameshift | **PGTPKGKRPV** | 2 (0.11) |
| **CCCGAACTCCCAAAGGGAAGATGGCCTGGTTT** | +1 frameshift | **PELPKGRWPG** | 1 (0.06) |
| **CCCGAACTCCCAAAGGGAAGTAAGGCCTGTTT** | +2 frameshift | **PELPKGSKAC** | 2 (0.11) |
| **CCCCGAAGCTCCCAAAGGGAAGAGGCCTGTTT** | +2 frameshift | **PRSSQREEAC** | 1 (0.06) |
| **CCCCGAACTCCCAAAGGGGAAGAGGCCTGTTT** | +2 frameshift | **PRTPKGEEAC** | 1 (0.06) |
| **CCCGAACTCCCCAAAGGGAAAGAGGCCTGTTT** | +2 frameshift | **PELPKGKEAC** | 1 (0.06) |
| **CCCCGAACTCCCAAAGGGAAGAAGGCCTGTTT** | +2 frameshift | **PRTPKGKKAC** | 1 (0.06) |
| **CCCGAAACTCCCAAAGGGAAGAGGCCATCGTTT** | insertion/substitution | **PETPKGKRPSF^†^** | 1 (0.06) |
| **CCCGAAACTCCCAAAGGGGAAGTAAGGCCTGTTT** | +1 frameshift | **PETPKGEVRPV** | 1 (0.06) |
| **CCCCGAACTCCCAAAGGGAAGGAAGGCCATCGTTT** | +1 frameshift | **PRTPKGKEGHR** | 1 (0.06) |
| **CCCGCAACTACCCGAAAAGGGCAAGAGGCCTTGTTT** | +2 frameshift | **PATTRKGQEALF^†^** | 1 (0.06) |
|  |  | Total no. reads: | 1778 |
|  |  | No. variants: | 39 [0.02] |

1. **H7N1 Passage 2**

| **HA_0_ cDNA nucleotide sequence^#^** | **Main mutational**  **effect** | **Translated**  **amino acids** | **No. of reads**  **(percentage)** |
| --- | --- | --- | --- |
| **CCCGAACTCCCAAAGGGAAGAGGCCTGTTT**^‡^ |  | **PELPKGRGLF^†^** | 2730 (95.15) |
| **CCCGAACTCC__AAGGGAAGAGGCCTGTTT** | +2 frameshift | **PELQGKRPV** | 4 (0.14) |
| **CCCGAACTCC_AAAGG_AAGAGGCCTGTTT** | +2 frameshift | **PELQRKRPV** | 1 (0.03) |
| **CCCGAACTCCAAC_GGAAGAGGCGTCGTTT** | +1 frameshift | **PELQRKRRR** | 11 (0.38) |
| **CCCGAACTCC_AAGGGAAGAGGCCTTGTTT** | +1 frameshift | **PELQGKRPC** | 1 (0.03) |
| **CCCGAACTCCCAAAGGAAAGAGGCCTGTTT** | substitution | **PELPKERGLF^†^** | 1 (0.03) |
| **CCCGAACTCCCAAAGGGAAAAGGCCTGTTT** | substitution | **PELPKGKGLF^†^** | 4 (0.14) |
| **CCCGAACTCCCAAAGGGAAGAGGCCCGTTT** | substitution | **PELPKGRGPF^†^** | 1 (0.03) |
| **CCCGAACCCCCAAAGGGAAGAGGCCTGTTT** | substitution | **PEPPKGRGLF^†^** | 3 (0.10) |
| **CCCGGACTCCCAAAGGGAAGAGGCCTGTTT** | substitution | **PGLPKGRGLF^†^** | 2 (0.07) |
| **CCCGAACTCCGAAAGGGAAGAGGCCTGTTT** | substitution | **PELRKGRGLF^†^** | 1 (0.03) |
| **CCCAAACTCCCAAAGGGAAGAGGCCTGTTT** | substitution | **PKLPKGRGLF^†^** | 4 (0.14) |
| **CCCGAACTCCCAAGGGGAAGAGGCCTGTTT** | substitution | **PELPRGRGLF^†^** | 5 (0.17) |
| **CCCGAAGCTCCCAAAGGGAAGAGGCCTGTTT** | +1 frameshift | **PEAPKGKRPV** | 1 (0.03) |
| **CCCGAACATCCCAAAGGGAAGAGGCCTGTTT** | +1 frameshift | **PEHPKGKRPV** | 1 (0.03) |
| **CCCGAACTCCCAAAGGGGAAGAGGCCTGTTT** | +1 frameshift | **PELPKGKRPV** | 16 (0.56) |
| **CCCGAACTCCCAAAAGGGAAGAGGCCTGTTT** | +1 frameshift | **PELPKGKRPV** | 1 (0.03) |
| **CCCGAACTCCCGAAAGGGAAGAGGCCTGTTT** | +1 frameshift | **PELPKGKRPV** | 2 (0.07) |
| **CCCGAACTCCCAAAGGGAAAGAGGCCTGTTT** | +1 frameshift | **PELPKGKRPV** | 1 (0.03) |
| **CCCGAACTCCCCAAAGGGAAGAGGCCTGTTT** | +1 frameshift | **PELPKGKRPV** | 2 (0.07) |
| **CCCGAACTCCCAAAGGGTAAGAGGCCTGTTT** | +1 frameshift | **PELPKGKRPV** | 2 (0.07) |
| **CCCGAACTCCCAAAGGGAATGAGGCCTGTTT** | +1 frameshift | **PELPKGMRPV** | 1 (0.03) |
| **CCCGAACTCCCAAAGGGAAGAGGCCATGTTT** | +1 frameshift | **PELPKGRGHV** | 1 (0.03) |
| **CCCGAACTCCCAAAGGGAAGAGGCCTCGTTT** | +1 frameshift | **PELPKGRGLV** | 2 (0.07) |
| **CCCGAACTCCCAAAGGGAAGAGGCCTTGTTT** | +1 frameshift | **PELPKGRGLV** | 4 (0.14) |
| **CCCGAACTCCCAAAGGGAAGAGGCCTAGTTT** | +1 frameshift | **PELPKGRGLV** | 1 (0.03) |
| **CCCGAACTCCCAAAGGGAAGAGGCCCTGTTT** | +1 frameshift | **PELPKGRGPV** | 2 (0.07) |
| **CCCGAACTCCCAAAGGGAAGAGGGCCTGTTT** | +1 frameshift | **PELPKGRGPV** | 10 (0.35) |
| **CCCGAACTCCCAAAGGGAAGGAGGCCTGTTT** | +1 frameshift | **PELPKGRRPV** | 5 (0.17) |
| **CCCGAACTCCCAAAGGGAAGAAGGCCTGTTT** | +1 frameshift | **PELPKGRRPV** | 2 (0.07) |
| **CCCGAACTCCCAAAGGGAAGACGGCCTGTTT** | +1 frameshift | **PELPKGRRPV** | 2 (0.07) |
| **CCCGAACTCCCAAAGGGAAGATGGCCTGTTT** | +1 frameshift | **PELPKGRWPV** | 3 (0.10) |
| **CCCGAACTCCCAAAGGGAAGCAGGCCTGTTT** | +1 frameshift | **PELPKGSRPV** | 5 (0.17) |
| **CCCGAATCTCCCAAAGGGAAGAGGCCTGTTT** | +1 frameshift | **PESPKGKRPV** | 3 (0.10) |
| **CCCGAAACTCCCAAAGGGAAGAGGCCTGTTT** | +1 frameshift | **PETPKGKRPV** | 9 (0.31) |
| **CCCGGAACTCCCAAAGGGAAGAGGCCTGTTT** | +1 frameshift | **PGTPKGKRPV** | 2 (0.07) |
| **CCCCGAACTCCCAAAGGGAAGAGGCCTGTTT** | +1 frameshift | **PRTPKGKRPV** | 2 (0.07) |
| **CCCGAACTCCCAAACGGGAAGAGGCCTGTTT** | +1 frameshift | **PELPNGKRPV** | 3 (0.10) |
| **CCCGAAGCTCCCAAAGGGAAGAAGGCCTGTTT** | +2 frameshift | **PEAPKGKKAC** | 1 (0.03) |
| **CCCGAACTCCCAAAGGGGAAGAGGCCTGGTTT** | +2 frameshift | **PELPKGKRPG** | 1 (0.03) |
| **CCCGAACTCCCAAAGGGGAAGGAGGCCTGTTT** | +2 frameshift | **PELPKGKEAC** | 1 (0.03) |
| **CCCGAACTCCCAAATGGGAAGAGGCCCTGTTT** | +2 frameshift | **PELPNGKRPC** | 1 (0.03) |
| **CCCGAACCTCCCAAAGGGAAGAGGGCCTGTTT** | +2 frameshift | **PEPPKGKRAC** | 1 (0.03) |
| **CCCGAACCTCCCAAAGGGAAGAGGCCTCGTTT** | +2 frameshift | **PEPPKGKRPR** | 1 (0.03) |
| **CCCGAAACTCCCAAAGGGAAGAGGCCTCGTTT** | +2 frameshift | **PETPKGKRPR** | 1 (0.03) |
| **CCCGAACCTCCCAAAGGGAAGAGGGCCTGTTT** | +2 frameshift | **PEPPKGKRAC** | 1 (0.03) |
| **CCCGAACCTCCCAAAGGGAAGAGGCCTCGTTT** | +2 frameshift | **PEPPKGKRPR** | 1 (0.03) |
| **CCCGAACTCCCAAACGGGAAGAGGCCGTCGTTT** | insertion/substitution | **PELPNGKRPSF^†^** | 1 (0.03) |
| **CCCGAACTCCCAAAGGGAAGGAGGACCGTGTTT** | insertion/substitution | **PELPKGRRTVF^†^** | 1 (0.03) |
| **CCCGAACTCCCAAAGGGAAGAGGGCCGTCGTTT** | insertion/substitution | **PELPKGRGPSF^†^** | 1 (0.03) |
| **CCCGAAACTCCCAAAGGGAAGAGGGACCTGTTT** | insertion/substitution | **PETPKGKRDLF^†^** | 1 (0.03) |
| **CCCGAAACTCCCAAAGGGAAGAGGTCCCTGTTT** | insertion/substitution | **PETPKGKRSLF^†^** | 1 (0.03) |
| **CCCGAACTCCCAAAGGGGAAGAGGGCCCTTGTTT** | +1 frameshift | **PELPKGKRALV** | 1 (0.03) |
| **CCCGAACTCCCCGAAAAGGGGAAGAGGCCTGTTT** | +1 frameshift | **PELPEKGKRPV** | 1 (0.03) |
| **CCCGAAACTCCCCAAAAGGGGAAGAGGGCCTGTTT** | +2 frameshift | **PETPQKGKRAC** | 1 (0.03) |
| **CCCGAACTCCCAAAGGGGAAGGTAGGGCCTCGTTT** | +2 frameshift | **PELPKGKVGPR** | 1 (0.03) |
|  |  | Total no. reads: | 2869 |
|  |  | No. variants: | 56 [0.02] |

1. **H7N1 Passage 3**

| **HA_0_ cDNA nucleotide sequence^#^** | **Main mutational**  **effect** | **Translated**  **amino acids** | **No. of reads**  **(percentage)** |
| --- | --- | --- | --- |
| **CCCGAACTCCCAAAGGGAAGAGGCCTGTTT**^‡^ |  | **PELPKGRGLF^†^** | 3910 (97.36) |
| **CCCGAACTCCCAAAGGGAA__GGCCTGTTT** | +2 frameshift | **PELPKGRPV** | 1 (0.025) |
| **CCCGAACTCC_AA_GGGAAGAGGCCTGTTT** | +2 frameshift | **PELQGKRPV** | 3 (0.07) |
| **CCCGAACTCC_AA_GGGAAGAGGCCGTGTTT** | +1 frameshift | **PELQGKRPC** | 1 (0.03) |
| **CCCGAACTTCCCAAAGGGAAGAGGCCTGTT** | +1 frameshift | **PELPKGKRPV** | 1 (0.03) |
| **CCCGAACATCCAAAGGGAAGAGGCCTGTTT** | substitution | **PEHPKGRGLF^†^** | 1 (0.03) |
| **CCCGAACTCCCAAAGGGAAAAGGCCTGTTT** | substitution | **PELPKGKGLF^†^** | 1 (0.03) |
| **CCCGAATTCCCAAAGGGAAGAGGCCTGTTT** | substitution | **PEFPKGRGLF^†^** | 1 (0.03) |
| **CCCGAACTCCCAAAGGGAAGAGGCCCGTTT** | substitution | **PELPKGRGPF^†^** | 2 (0.05) |
| **CCCGAACTCCCAAGGGGAAGAGGCCTGTTT** | substitution | **PELPRGRGLF^†^** | 7 (0.17) |
| **CCCGAACTCCCAACGGGAAGAGGCCTGTTT** | substitution | **PELPTGRGLF^†^** | 2 (0.05) |
| **CCCGAAGTCCCAAAGGGAAGAGGCCTGTTT** | substitution | **PEVPKGRGLF^†^** | 2 (0.05) |
| **CCCGGACTCCCAAAGGGAAGAGGCCTGTTT** | substitution | **PGLPKGRGLF^†^** | 1 (0.03) |
| **CCCGAACTCCAAAAGGGAAGAGGCCTGTTT** | substitution | **PELQKGRGLF^†^** | 2 (0.05) |
| **CCCGAACTCCAACGGAAGGAGGGCCTGTTT** | substitution | **PELQRKEGLF^†^** | 1 (0.03) |
| **CCCGAACTCTCAAAGGGAAGAGGCCTGTTT** | substitution | **PELSKGRGLF^†^** | 1 (0.03) |
| **CCCGAACCCCCAAAGGGAAGAGGCCTGTTT** | substitution | **PEPPKGRGLF^†^** | 1 (0.03) |
| **CCCGGAACTCCCAAAGGGAAGAGGCCTGTTT** | +1 frameshift | **PGTPKGKRPV** | 1 (0.03) |
| **CCCGAACATCCCAAAGGGAAGAGGCCTGTTT** | +1 frameshift | **PEHPKGKRPV** | 2 (0.05) |
| **CCCGAAGCTCCCAAAGGGAAGAGGCCTGTTT** | +1 frameshift | **PEAPKGKRPV** | 5 (0.12) |
| **CCCGAACTCCCGAAAGGGAAGAGGCCTGTTT** | +1 frameshift | **PELPKGKRPV** | 2 (0.05) |
| **CCCGAACTCCCAAAGGGGAAGAGGCCTGTTT** | +1 frameshift | **PELPKGKRPV** | 13 (0.32) |
| **CCCGAACTCCCAAAGGGTAAGAGGCCTGTTT** | +1 frameshift | **PELPKGKRPV** | 1 (0.03) |
| **CCCGAACTCCCAAAGGGAAAGAGGCCTGTTT** | +1 frameshift | **PELPKGKRPV** | 3 (0.07) |
| **CCCGAACTCCCCAAAGGGAAGAGGCCTGTTT** | +1 frameshift | **PELPKGKRPV** | 3 (0.07) |
| **CCCGAACTCCCAAAAGGGAAGAGGCCTGTTT** | +1 frameshift | **PELPKGKRPV** | 1 (0.03) |
| **CCCGAACTCCCAAAGGGAAGAGGCCATGTTT** | +1 frameshift | **PELPKGRGHV** | 3 (0.07) |
| **CCCGAACTCCCAAAGGGAAGAGGCCTTGTTT** | +1 frameshift | **PELPKGRGLV** | 3 (0.07) |
| **CCCGAACTCCCAAAGGGAAGAGGCCTGGTTT** | +1 frameshift | **PELPKGRGLV** | 3 (0.07) |
| **CCCGAACTCCCAAAGGGAAGAGGCCTCGTTT** | +1 frameshift | **PELPKGRGLV** | 1 (0.03) |
| **CCCGAACTCCCAAAGGGAAGAGGACCTGTTT** | +1 frameshift | **PELPKGRGPV** | 5 (0.12) |
| **CCCGAACTCCCAAAGGGAAGAGGCCGTGTTT** | +1 frameshift | **PELPKGRGRV** | 3 (0.07) |
| **CCCGAACTCCCAAAGGGAAGACGGCCTGTTT** | +1 frameshift | **PELPKGRRPV** | 2 (0.05) |
| **CCCGAACTCCCAAAGGGAAGATGGCCTGTTT** | +1 frameshift | **PELPKGRWPV** | 1 (0.03) |
| **CCCGAACTCCCAAAGGGAAGCAGGCCTGTTT** | +1 frameshift | **PELPKGSRPV** | 4 (0.10) |
| **CCCGAACTCCCAAAGGGAACGAGGCCTGTTT** | +1 frameshift | **PELPKGTRPV** | 3 (0.07) |
| **CCCGAACCTCCCAAAGGGAAGAGGCCTGTTT** | +1 frameshift | **PEPPKGKRPV** | 1 (0.03) |
| **CCCGAATCTCCCAAAGGGAAGAGGCCTGTTT** | +1 frameshift | **PESPKGKRPV** | 1 (0.03) |
| **CCCGAACTCCCAAATGGGAAGAGGCCTGTTT** | +1 frameshift | **PELPNGKRPV** | 2 (0.05) |
| **CCCGAAACTCCCAAAGGGAAGAGGCCTGTTT** | +1 frameshift | **PETPKGKRPV** | 7 (0.17) |
| **CCCGAACTCCCAAAGGGGAAGAGGGCCTGTTT** | +2 frameshift | **PELPKGKRAC** | 2 (0.05) |
| **CCCGAATCTCCCAAAGGGAAGAGGCCTTGTTT** | +2 frameshift | **PESPKGKRPC** | 1 (0.03) |
| **CCCGAACTCCCCAAAAGGGAAGAGGCCTGTTT** | +2 frameshift | **PELPKREEAC** | 1 (0.03) |
| **CCCGAACTCCCAAACGGGAAGAGGCCATGTTT** | +2 frameshift | **PELPNGKRPC** | 1 (0.03) |
| **CCCGAAACTCCCAAAGGGAAGAGGGCCTGTTT** | +2 frameshift | **PETPKGKRAC** | 1 (0.03) |
| **CCCGAAACTCCCAAAGGGAAGAGGCCTTGTTT** | +2 frameshift | **PETPKGKRPC** | 1 (0.03) |
| **CCCGAACTCCCAAAGGGAAGAGGACCGTGTTT** | +2 frameshift | **PELPKGRGPC** | 1 (0.03) |
|  |  | Total no. reads: | 4016 |
|  |  | No. variants: | 47 [0.01] |

1. **H7N1 Passage 4**

| **HA_0_ cDNA nucleotide sequence^#^** | **Main mutational**  **effect** | **Translated**  **amino acids** | **No. of reads**  **(percentage)** |
| --- | --- | --- | --- |
| **CCCGAACTCCCAAAGGGAAGAGGCCTGTTT**^‡^ |  | **PELPKGRGLF^†^** | 60 (100) |

1. **H7N1 Passage 5**

| **HA_0_ cDNA nucleotide sequence^#^** | **Main mutational**  **effect** | **Translated**  **amino acids** | **No. of reads**  **(percentage)** |
| --- | --- | --- | --- |
| **CCCGAACTCCCAAAGGGAAGAGGCCTGTTT**^‡^ |  | **PELPKGRGLF^†^** | 72 (97.23) |
| **CCCGAATTCCCAAAGGGAAGAGGCCTGTTT** | substitution | **PEFPKGRGLF^†^** | 1 (1.35) |
| **CCCGAACTCCCCAAAGGGAAGAGGCCTGTTT** | +1 frameshift | **PELPKGKRPV** | 1 (1.35) |
|  |  | Total no. reads: | 74 |
|  |  | No. variants: | 3 [0.04] |

1. **H7N1 Passage 6**

| **HA_0_ cDNA nucleotide sequence^#^** | **Main mutational**  **effect** | **Translated**  **amino acids** | **No. of reads**  **(percentage)** |
| --- | --- | --- | --- |
| **CCCGAACTCCCAAAGGGAAGAGGCCTGTTT**^‡^ |  | **PELPKGRGLF^†^** | 985 (95.63) |
| **CCCGAACTCC_AA_GGGAAGAGGCCTGTTT** | +2 frameshift | **PELQGKRPV** | 1 (0.10) |
| **CCCGAACTCCCAAAGGGAAGAGGCCTGGTT** | substitution | **PELPKGRGLV^†^** | 1 (0.10) |
| **CCCGAACTCTCAAAGGGAAGAGGCCTGTTT** | substitution | **PELSKGRGLF^†^** | 1 (0.10) |
| **CCCGAACTCCCAAAGGGAAGAGACCTGTTT** | substitution | **PELPKGRDLF^†^** | 1 (0.10) |
| **CCCGAACTCCCAAAGGGAAGAGGCCTGCTT** | substitution | **PELPKGRGLL^†^** | 1 (0.10) |
| **CCCGAACTCCCAAGGGGAAGAGGCCTGTTT** | substitution | **PELPRGRGLF^†^** | 3 (0.29) |
| **CCCGAACTCCCAAAGGGAAGAGGCCCGTTT** | substitution | **PELPKGRGPF^†^** | 1 (0.10) |
| **CCCGAACTCCCAAAGGGTAAGAGGCCTGTTT** | +1 frameshift | **PELPKGKRPV** | 1 (0.10) |
| **CCCGAACTCCCCAAAGGGAAGAGGCCTGTTT** | +1 frameshift | **PELPKGKRPV** | 1 (0.10) |
| **CCCGAACTCCCAAAGGGGAAGAGGCCTGTTT** | +1 frameshift | **PELPKGKRPV** | 3 (0.29) |
| **CCCGAACTCCCGAAAGGGAAGAGGCCTGTTT** | +1 frameshift | **PELPKGKRPV** | 1 (0.10) |
| **CCCGAACTCCCAAAGGGAAAGAGGCCTGTTT** | +1 frameshift | **PELPKGKRPV** | 1 (0.10) |
| **CCCGAACTCCCAAAAGGGAAGAGGCCTGTTT** | +1 frameshift | **PELPKGKRPV** | 1 (0.10) |
| **CCCGAACTCCCAAAGGGAAGAGGCCTCGTTT** | +1 frameshift | **PELPKGRGLV** | 2 (0.19) |
| **CCCGAACTCCCAAAGGGAAGAGGACCTGTTT** | +1 frameshift | **PELPKGRGPV** | 2 (0.19) |
| **CCCGAACTCCCAAAGGGAAGAGGGCCTGTTT** | +1 frameshift | **PELPKGRGPV** | 3 (0.29) |
| **CCCGAACTCCCAAAGGGAAGGAGGCCTGTTT** | +1 frameshift | **PELPKGRRPV** | 2 (0.19) |
| **CCCGAACTCCCAAAGGGAAGATGGCCTGTTT** | +1 frameshift | **PELPKGRWPV** | 1 (0.10) |
| **CCCGAACTCCCAA_GGGAACGAGGCCTTGTTT** | +1 frameshift | **PELPRERGLV** | 1 (0.10) |
| **CCCGAACTCCGAAAGGGAAGAGGCCTTGTTT** | +1 frameshift | **PELRKGRGLV** | 1 (0.10) |
| **CCCGAACGTCCCAAAGGGAAGAGGCCTGTTT** | +1 frameshift | **PERPKGKRPV** | 1 (0.10) |
| **CCCGAAGCTCCCAAAGGGAAGAGGCCTGGTTT** | +2 frameshift | **PEAPKGKRPG** | 1 (0.10) |
| **CCCGAACTCCCGAAAGGGAAGGAGGCCTGTTT** | +2 frameshift | **PELPKGKEAC** | 4 (0.39) |
| **CCCGAAGCTCCCAAAGGGAAGATGGCCTGTTT** | +2 frameshift | **PEAPKGKMAC** | 1 (0.10) |
| **CCCGAAGCTCCCAAAGGGAAGAGGGCCTGTTT** | +2 frameshift | **PEAPKGKRAC** | 1 (0.10) |
| **CCCGAAACTCCCAAAGGGGAAGAGGCCTGTTT** | +2 frameshift | **PETPKGEEAC** | 1 (0.10) |
| **CCCGAAACTCCCAAACGGGAAGAGGCCTGTTT** | +2 frameshift | **PETPKREEAC** | 1 (0.10) |
| **CCCGAACTCCCAAAGGGGAAGAAGGCCTGTTT** | +2 frameshift | **PELPKGKKAC** | 1 (0.10) |
| **CCCGAACTCCCAAACGGGAAGAGGGCCTGTTT** | +2 frameshift | **PELPNGKRAC** | 1 (0.10) |
| **CCCGAACTCCCAAAGGGGAAGGAGGCCTTGTTT** | insertion/substitution | **PELPKGKEALF^†^** | 1 (0.10) |
| **CCCGAACTCCCCAAAGGGGAAAGAGGGCCTGTTT** | +1 frameshift | **PELPKGERGPV** | 1 (0.10) |
| **CCCGAACTCCCGAAACGGGAAGAGGACCTTGTTT** | +1 frameshift | **PELPKREEDLV** | 1 (0.10) |
| **CCCGAAACTCCCCAAAAGGGGAAGAGGGCCCTGTTT** | insertion/substitution | **PETPQKGKRALF^†^** | 1 (0.10) |
|  |  | Total no. reads: | 1030 |
|  |  | No. variants: | 34 [0.03] |

1. **H7N1 Passage 7**

| **HA_0_ cDNA nucleotide sequence^#^** | **Main mutational**  **effect** | **Translated**  **amino acids** | **No. of reads**  **(percentage)** |
| --- | --- | --- | --- |
| **CCCGAACTCCCAAAGGGAAGAGGCCTGTTT**^‡^ |  | **PELPKGRGLF^†^** | 2232 (95.75) |
| **CCCGAACTCC_AAGGGAAGAAGGCCTGTTT** | +1 frameshift | **PELQGKKAC** | 1 (0.04) |
| **CCCGAATTCCCAAAGGGAAGAGGCCTGTTT** | substitution | **PEFPKGRGLF^†^** | 2 (0.09) |
| **CCCGAACATCCCAAGGGAAGAGGCCTGTTT** | substitution | **PEHPKGRGLF^†^** | 1 (0.04) |
| **CCCGAACTCCCAAAGGAAAGAGGCCTGTTT** | substitution | **PELPKERGLF^†^** | 1 (0.04) |
| **CCCGAACTCCCTAAAGGGAAGAGGCCGTTT** | substitution | **PELPKGKRPF^†^** | 1 (0.04) |
| **CCCGAACTCCCAAAGGGAAAAGGCCTGTTT** | substitution | **PELPKGKGLF^†^** | 2 (0.09) |
| **CCCGAACTCCCAAAGAGAAGAGGCCTGTTT** | substitution | **PELPKRRGLF^†^** | 3 (0.13) |
| **CCCGAACTCCCAA_GGGAACGAGGCCTGTTT** | substitution | **PELPRERGLF^†^** | 2 (0.09) |
| **CCCGAACTCCCAAGGGGAAGAGGCCTGTTT** | substitution | **PELPRGRGLF^†^** | 1 (0.04) |
| **CCCGAACTCCAACGGAAGGAAGGCCTGTTT** | substitution | **PELQRKEGLF^†^** | 1 (0.04) |
| **CCCGAACCCCCAAAGGGAAGAGGCCTGTTT** | substitution | **PEPPKGRGLF^†^** | 3 (0.13) |
| **CCCGAATCCCCAAAGGGAAGAGGCCTGTTT** | substitution | **PESPKGRGLF^†^** | 1 (0.04) |
| **CCCGAAACTCC_AAAGGGAAGAGGCCTGTTT** | substitution | **PETPKGRGLF^†^** | 1 (0.04) |
| **CCCGGACTCCCAAAGGGAAGAGGCCTGTTT** | substitution | **PGLPKGRGLF^†^** | 2 (0.09) |
| **CCCGAACTCCCAAAGGGAAGAGGCCTGATT** | substitution | **PELPKGRGLI^†^** | 1 (0.04) |
| **CCCGAACTCCCAAAGGGAAGAGGCCTGCTT** | substitution | **PELPKGRGLL^†^** | 1 (0.04) |
| **CCCGAACTCCCAAAGGGAAGAGGCCTTGTT** | substitution | **PELPKGRGLV^†^** | 2 (0.09) |
| **CCCGAACTCCCAAAGGGAAGAGGCCTGGTT** | substitution | **PELPKGRGLV^†^** | 3 (0.13) |
| **CCCGAACTCCCAAAGGGAAGAGGCCCGTTT** | substitution | **PELPKGRGPF^†^** | 2 (0.09) |
| **CCCGAACTCCCAAAGGGAAGAGGCCCTGTTT** | +1 frameshift | **PELPKGRGPV** | 3 (0.13) |
| **CCCGAACTCCCAAAGGGAAGAGGACCTGTTT** | +1 frameshift | **PELPKGRGPV** | 3 (0.13) |
| **CCCGAACTCCCAAAGGGAAGAGGTCCTGTTT** | +1 frameshift | **PELPKGRGPV** | 2 (0.09) |
| **CCCGAACTCCCAAAGGGAAGAGGGCCTGTTT** | +1 frameshift | **PELPKGRGPV** | 6 (0.26) |
| **CCCGAACTCCCAAAGGGAAGAGGCCAGGTTT** | +1 frameshift | **PELPKGRGQV** | 1 (0.04) |
| **CCCGAACTCCCAAAGGGAAGAGGCCGTGTTT** | +1 frameshift | **PELPKGRGRV** | 3 (0.13) |
| **CCCGAACTCCCAAAGGGAAGACGGCCTGTTT** | +1 frameshift | **PELPKGRRPV** | 2 (0.09) |
| **CCCGAACTCCCAAAGGGAAGATGGCCTGTTT** | +1 frameshift | **PELPKGRWPV** | 1 (0.04) |
| **CCCGAAGCTCCCAAAGGGAAGAGGCCTGTTT** | +1 frameshift | **PEAPKGKRPV** | 4 (0.17) |
| **CCCGAAACTCCCAAAGGGAAGAGGCCTGTTT** | +1 frameshift | **PETPKGKRPV** | 7 (0.30) |
| **CCCGAACTCCCAAAGGGTAAGAGGCCTGTTT** | +1 frameshift | **PELPKGKRPV** | 2 (0.09) |
| **CCCGAACTCCCAAAGGGGAAGAGGCCTGTTT** | +1 frameshift | **PELPKGKRPV** | 7 (0.30 |
| **CCCGAACTCCCAAAAGGGAAGAGGCCTGTTT** | +1 frameshift | **PELPKGKRPV** | 2 (0.09) |
| **CCCGAACTCCCCAAAGGGAAGAGGCCTGTTT** | +1 frameshift | **PELPKGKRPV** | 4 (0.17) |
| **CCCGAACTCCCAAAGGGAATGAGGCCTGTTT** | +1 frameshift | **PELPKGMRPV** | 1 (0.04) |
| **CCCGGACTCCCAAAGGGAAGAGGACCTGTTT** | +1 frameshift | **PGLPKGRGPV** | 1 (0.04) |
| **CCCGAACTCCCAAACGGGAAGAGGCCTGTTT** | +1 frameshift | **PELPNGKRPV** | 4 (0.17) |
| **CCCGAATCTCCCAAAGGGAAGAGGCCTGTTT** | +1 frameshift | **PESPKGKRPV** | 1 (0.04) |
| **CCCGGAACTCCCAAAGGGAAGAGGCCTCGTTT** | +2 frameshift | **PGTPKGKRPR** | 1 (0.04) |
| **CCCGAACTCCCCAAAGGGAAAGAGGCCTGTTT** | +2 frameshift | **PELPKGKEAC** | 1 (0.04) |
| **CCCGAACTCCCAAAGGGGAAGAGGGCCTGTTT** | +2 frameshift | **PELPKGKRAC** | 2 (0.09) |
| **CCCGAACTCCCAAAGGGAAGAGCGGCCTGTTT** | +2 frameshift | **PELPKGRAAC** | 1 (0.04) |
| **CCCGAACTCCCAAAGGGAAGAAGGGCCTGTTT** | +2 frameshift | **PELPKGRRAC** | 1 (0.04) |
| **CCCGAAACTCCCAAAGGGAAAGAGGCCTGTTT** | +2 frameshift | **PETPKGKEAC** | 1 (0.04) |
| **CCCGAACTCCCAAGGGTAATGAGGCCATGTTT** | +2 frameshift | **PELPRVMRPC** | 1 (0.04) |
| **CCCGAACTCCCAAACGGGAAGAGGCCGTGTTT** | +2 frameshift | **PELPNGKRPC** | 1 (0.04) |
| **CCCGAACTCCCAAAGGGGAAGAAGGGCCTGTTT** | insertion/substitution | **PELPKGKKGLF^†^** | 1 (0.04) |
| **CCCGAACTCCCCAAAAGGGGAAGAGGCCTGTTT** | insertion/substitution | **PELPKRGRGLF^†^** | 1 (0.04) |
| **CCCGAAACTCCCCAAAAGGGAAGAGGCCTGTTT** | insertion/substitution | **PETPQKGRGLF^†^** | 1 (0.04) |
| **CCCGAAAGCTTCCCAAAGGGAAGAAGGCCTGTTT** | +1 frameshift | **PESFPKGRRPV** | 1 (0.04) |
| **CCCGGAACTCCCAAAGGGAAGGAAGGCCTTGTTT** | +1 frameshift | **PGTPKGKEGLV** | 1 (0.04) |
|  |  | Total no. reads: | 2331 |
|  |  | No. variants: | 51 [0.02] |

1. **H7N1 Passage 11**

| **HA_0_ cDNA nucleotide sequence^#^** | **Main mutational**  **effect** | **Translated**  **amino acids** | **No. of reads**  **(percentage)** |
| --- | --- | --- | --- |
| **CCCGAACTCCCAAAGGGAAGAGGCCTGTTT**^‡^ |  | **PELPKGRGLF** | 3406 (93.96) |
| **CCCGAACTCCCAAAGGGAA__GGCCTGTTT** | +2 frameshift | **PELPKGRPV** | 2 (0.06) |
| **CCCGA_CTCCCAA_GGGAAGAGGCCTGTTT** | +2 frameshift | **PDSQGKRPV** | 1 (0.03) |
| **CCCGAACTCC_AAAGG_AAGAGGCCTGTTT** | +2 frameshift | **PELQRKRPV** | 1 (0.03) |
| **CCCGAACTCC_AACGGAAGGAGGCCTGTTT** | +1 frameshift | **PELQRKEAC** | 2 (0.06) |
| **CCCGAATTCCCAAAGGGAAGAGGCCTGTTT** | substitution | **PEFPKGRGLF^†^** | 1 (0.03) |
| **CCCGAAATCCCAAAGGGAAGAGGCCTGTTT** | substitution | **PEIPKGRGLF^†^** | 2 (0.06) |
| **CCCGAACTCCCAAGGGGAAGAGGCCTGTTT** | substitution | **PELPRGRGLF^†^** | 3 (0.08) |
| **CCCGAACTCCCAAAGGGAAGAGGCCGGTTT** | substitution | **PELPKGRGRF^†^** | 2 (0.06) |
| **CCCGAACTCCCAAAGGGAAGAGGCATGTTT** | substitution | **PELPKGRGMF^†^** | 2 (0.06) |
| **CCCGAACTCCCAAAGGGAAGAGGCCCGTTT** | substitution | **PELPKGRGPF^†^** | 4 (0.11) |
| **CCCGAACTCCAACGGAAGGAGGCGTCGTTT** | substitution | **PELQRKEASF^†^** | 1 (0.03) |
| **CCCGAACTCCAACGGAAGGAAGGCCTGTTT** | substitution | **PELQRKEGLF^†^** | 1 (0.03) |
| **CCCGAACTCCAACGGAAGAGGCCGTCGTTT** | substitution | **PELQRKRPSF^†^** | 1 (0.03) |
| **CCCGAACCCCCAAAGGGAAGAGGCCTGTTT** | substitution | **PEPPKGRGLF^†^** | 4 (0.11) |
| **CCCGAAACTCCCAAGGGAAGAGGCCTGTTT** | substitution | **PETPKGRGLF^†^** | 1 (0.03) |
| **CCCGGACTCCCAAAGGGAAGAGGCCTGTTT** | substitution | **PGLPKGRGLF^†^** | 3 (0.08) |
| **CC_GAAGCTCCCAAAGGGAAGAGGCCTGTTT** | substitution | **PKLPKGRGLF^†^** | 1 (0.03) |
| **CCCGAACATCCCAAAGGGAAGAGGCCTGTTT** | +1 frameshift | **PEHPKGKRPV** | 11 (0.30) |
| **CCCGAAGCTCCCAAAGGGAAGAGGCCTGTTT** | +1 frameshift | **PEAPKGKRPV** | 6 (0.17) |
| **CCCGCAACTCCCAAAGGGAAGAGGCCTGTTT** | +1 frameshift | **PATPKGKRPV** | 1 (0.03) |
| **CCCGAACTCCCAAAGGGGAAGAGGCCTATTT** | +1 frameshift | **PELPKGKRPI** | 1 (0.03) |
| **CCCGAACTCCCAAAGGGGAAGAGGCCTGTTT** | +1 frameshift | **PELPKGKRPV** | 25 (0.69) |
| **CCCGAACTCCCAAAAGGGAAGAGGCCTGTTT** | +1 frameshift | **PELPKGKRPV** | 7 (0.19) |
| **CCCGAACTACCCAAAGGGAAGAGGCCTGTTT** | +1 frameshift | **PELPKGKRPV** | 1 (0.03) |
| **CCCGAACTCCCCAAAGGGAAGAGGCCTGTTT** | +1 frameshift | **PELPKGKRPV** | 2 (0.06) |
| **CCCGAACTCCCAAAGGGAAAGAGGCCTGTTT** | +1 frameshift | **PELPKGKRPV** | 3 (0.08) |
| **CCCGAACTCCCAAAGGGTAAGAGGCCTGTTT** | +1 frameshift | **PELPKGKRPV** | 2 (0.06) |
| **CCCGAACTCCCGAAAGGGAAGAGGCCTGTTT** | +1 frameshift | **PELPKGKRPV** | 1 (0.03) |
| **CCCGAACTCCCAAAGGGAATGAGGCCTGTTT** | +1 frameshift | **PELPKGMRPV** | 1 (0.03) |
| **CCCGAACTCCCAAAGGGAAGAGGCCATGTTT** | +1 frameshift | **PELPKGRGHV** | 2 (0.06) |
| **CCCGAACTCCCAAAGGGAAGAGGCCTGATTT** | +1 frameshift | **PELPKGRGLI** | 7 (0.19) |
| **CCCGAACTCCCAAAGGGAAGAGGCCTGGTTT** | +1 frameshift | **PELPKGRGLV** | 1 (0.03) |
| **CCCGAACTCCCAAAGGGAAGAGGCCTCGTTT** | +1 frameshift | **PELPKGRGLV** | 7 (0.19) |
| **CCCGAACTCCCAAAGGGAAGAGGCCTTGTTT** | +1 frameshift | **PELPKGRGLV** | 3 (0.08) |
| **CCCGAACTCCCAAAGGGAAGAGGGCCTGTTT** | +1 frameshift | **PELPKGRGPV** | 11 (0.30) |
| **CCCGAACTCCCAAAGGGAAGAGGTCCTGTTT** | +1 frameshift | **PELPKGRGPV** | 1 (0.03) |
| **CCCGAACTCCCAAAGGGAAGAGGCCCTGTTT** | +1 frameshift | **PELPKGRGPV** | 2 (0.06) |
| **CCCGAACTCCCAAAGGGAAGAGGACCTGTTT** | +1 frameshift | **PELPKGRGPV** | 7 (0.19) |
| **CCCGAACTCCCAAAGGGAAGAGGCCGTGTTT** | +1 frameshift | **PELPKGRGRV** | 8 (0.22) |
| **CCCGAACTCCCAAAGGGAAGGAGGCCTGTTT** | +1 frameshift | **PELPKGRRPV** | 5 (0.14) |
| **CCCGAACTCCCAAAGGGAAGACGGCCTGTTT** | +1 frameshift | **PELPKGRRPV** | 2 (0.06) |
| **CCCGAACTCCCAAAGGGAAGCAGGCCTGTTT** | +1 frameshift | **PELPKGSRPV** | 3 (0.08) |
| **CCCGAACTCCCAAAGGGAACGAGGCCTGTTT** | +1 frameshift | **PELPKGTRPV** | 3 (0.08) |
| **CCCGAACTCCCAAACGGGAAGAGGCCTGTTT** | +1 frameshift | **PELPNGKRPV** | 7 (0.19) |
| **CCCGGAACTCCCAAAGGGAAGAGGCCTGTTT** | +1 frameshift | **PGTPKGKRPV** | 1 (0.03) |
| **CCCGAACTCCAAACGGGAAGAGGCGTCGTTT** | +1 frameshift | **PELQTGRGVV** | 1 (0.03) |
| **CCCGAACCTCCCAAAGGGAAGAGGCCTGTTT** | +1 frameshift | **PEPPKGKRPV** | 2 (0.06) |
| **CCCGAAACTCCCAAAGGGAAGAGGCCTGTTT** | +1 frameshift | **PETPKGKRPV** | 15 (0.41) |
| **CCCGAACATCCCAAACGGGAAGAGGCCTGTTT** | +2 frameshift | **PEHPKREEAC** | 1 (0.03) |
| **CCCGAACTCCCAAAGGGTAAGAGGTCCTGTTT** | +2 frameshift | **PELPKGKRSC** | 1 (0.03) |
| **CCCGAACTCCCAAAGGGGAAGAGGACCTGTTT** | +2 frameshift | **PELPKGKRTC** | 1 (0.03) |
| **CCCGAACTCCCAAAGGGAATGAGGCCTTGTTT** | +2 frameshift | **PELPKGMRPC** | 1 (0.03) |
| **CCCGAACTCCCAAAGGGAAGAGGCCTCGATTT** | +2 frameshift | **PELPKGRGLD** | 1 (0.03) |
| **CCCGAACTCCCAAAGGGGAAGAGGGCCTGTTT** | +2 frameshift | **PELPKGKRAC** | 1 (0.03) |
| **CCCGAACTCCCCAAAGGGAAGAGGCCTTGTTT** | +2 frameshift | **PELPKGKRPC** | 2 (0.06) |
| **CCCGAACTCCCAAAGGGGAAGAGGCCTGGTTT** | +2 frameshift | **PELPKGKRPG** | 3 (0.08) |
| **CCCGAACTCCCAAAGGGAAGAGGCCGTCGTTT** | +2 frameshift | **PELPKGRGRR** | 1 (0.03) |
| **CCCGAAGCTCCCAAAGGGAAGAGGTCCTGTTT** | +2 frameshift | **PEAPKGKRSC** | 1 (0.03) |
| **CCCGAAGCTCCCAAAGGGAAGAGGCCTTGTTT** | +2 frameshift | **PEAPKGKRPC** | 1 (0.03) |
| **CCCGAACTCCCAAAGGGAAGAGGGCCGTGTTT** | +2 frameshift | **PELPKGRGPC** | 1 (0.03) |
| **CCCGAACTCCCAAAGGGAAGAGGCCCGTGTTT** | +2 frameshift | **PELPKGRGPC** | 2 (0.06) |
| **CCCGAACTCCCAAAGGGAAGTAGGCCCTGTTT** | +2 frameshift | **PELPKGSRPC** | 1 (0.03) |
| **CCCGAACTCCCAAACGGGAAGAGGCCTTGTTT** | +2 frameshift | **PELPNGKRPC** | 2 (0.06) |
| **CCCGAACTCCCAAACGGGAAGAGGTCCTGTTT** | +2 frameshift | **PELPNGKRSC** | 1 (0.03) |
| **CCCGAACCTCCCAAAGGGAAGAGGCCCTGTTT** | +2 frameshift | **PEPPKGKRPC** | 1 (0.03) |
| **CCCGAAACTCCCAAAGGGAAAGAGGCCTGTTT** | +2 frameshift | **PETPKGKEAC** | 1 (0.03) |
| **CCCGAAACTCCCAAAGGGAAGAGGGCCTGTTT** | +2 frameshift | **PETPKGKRAC** | 2 (0.06) |
| **CCCGGAACTCCCAAAAGGGAAGAGGCCTGTTT** | +2 frameshift | **PGTPKREEAC** | 1 (0.03) |
| **CCCGAAACTCCCAAAGGGAAGAGGACCTGTTT** | +2 frameshift | **PETPKGKRTC** | 1 (0.03) |
| **CCCGAACTCCCCAAAGGGAAAGAGGCCTCGTTT** | insertion/substitution | **PELPKGKEASF^†^** | 1 (0.03) |
| **CCCGAACATCCCAAAGGGAAGAGGACCTGGTTT** | insertion/substitution | **PEHPKGKRTWF^†^** | 1 (0.03) |
| **CCCGAACTCCCAAAGGGAAGAAGGACCGTGTTT** | insertion/substitution | **PELPKGRRTVF^†^** | 1 (0.03) |
| **CCCGAACTCCCAAAGGGAAGAGGGACCATGTTT** | insertion/substitution | **PELPKGRGTMF^†^** | 1 (0.03) |
| **CCCGAACTCCCAAAGGGAAGAGGGCCTTGTTTT** | insertion/substitution | **PELPKGRGPCF^†^** | 1 (0.03) |
| **CCCGAAACTCCCAAAGGGAAGACGGCCGTGTTT** | insertion/substitution | **PETPKGKTAVF^†^** | 1 (0.03) |
| **CCCGAACTCCCAAAAGGGAAAGAGGCACCTGTTT** | +1 frameshift | **PELPKGKEAPV** | 1 (0.03) |
| **CCCGAAACTCCCAAAAGGGGAAGAAGGCCTGTTT** | +1 frameshift | **PETPKRGRRPV** | 1 (0.03) |
| **CCCGAACTCCCAAAGGGAAGAAGGGACCGTGTTT** | +1 frameshift | **PELPKGRRDRV** | 1 (0.03) |
| **CCCGAACTCCCAAAAGGGAAGGAGGACCGTAGTTT** | +2 frameshift | **PELPKGKEDRS** | 1 (0.03) |
| **CCCGAACATCCCAAATCGGGGAAAGAGGCCCTGTTT** | insertion/substitution | **PEHPKSGKEALF^†^** | 1 (0.03) |
|  |  | Total no. reads: | 3625 |
|  |  | No. variants: | 81 [0.02] |

1. **H7N1 Passage 15**

| **HA_0_ cDNA nucleotide sequence^#^** | **Main mutational**  **effect** | **Translated**  **amino acids** | **No. of reads**  **(percentage)** |
| --- | --- | --- | --- |
| **CCCGAACTCCCGAAGGGAAGAGGCCTGTTT**^‡^ |  | **PELPKGRGLF^†^** | 12985 (94.91) |
| **CCCGA_CTCCC_AAGGGAAGAGGCCTGTTT** | +2 frameshift | **PDSQGKRPV** | 1 (0.01) |
| **CCCGA_CTCCC_AAAGGAAGAGGCCTGTTT** | +2 frameshift | **PDSQRKRPV** | 1 (0.01) |
| **CCCGAACTCCCAAAGGGAA__GGCCTGTTT** | +2 frameshift | **PELPKGRPV** | 6 (0.04) |
| **CCCGAACTCCCAAAG__AACCTCCGACTTT** | +2 frameshift | **PELPKNLRL** | 2 (0.01) |
| **CCCGAACTCC_AA_GGGAAGAGGCCTGTTT** | +2 frameshift | **PELQGKRPV** | 2 (0.01) |
| **CCCGAACTCCCAA_GG_AAGAGGCCTGTTT** | +2 frameshift | **PELPRKRPV** | 4 (0.03) |
| **CCCGAACTCC_AAAGG_AAGAGGCCTGTTT** | +2 frameshift | **PELQRKRPV** | 3 (0.02) |
| **CCCGAACTCC_AAGGGAAGAGGCCTGCTTT** | +1 frameshift | **PELQGKRPA** | 1 (0.01) |
| **CCCGAACTCC_AAGGGAAGAGGCCTGATTT** | +1 frameshift | **PELQGKRPD** | 1 (0.01) |
| **CCCGAACTCCCAAAGGGAAAAGGCCTGTTT** | substitution | **PELPKGKGLF^†^** | 5 (0.04) |
| **CCCGGACTCCCAAAGGGAAGAGGCCTGTTT** | substitution | **PGLPKGRGLF^†^** | 3 (0.02) |
| **CCCGAACACCCAAAGGGAAGAGGCCTGTTT** | substitution | **PEHPKGRGLF^†^** | 2 (0.01) |
| **CCCGACCTCCCAAAGGGAAGAGGCCTGTTT** | substitution | **PDLPKGRGLF^†^** | 1 (0.01) |
| **CCCGAAATCCCAAAGGGAAGAGGCCTGTTT** | substitution | **PEIPKGRGLF^†^** | 1 (0.01) |
| **CCCGAACTCCTAAAGGGAAGAGGCCTGTTT** | substitution | **PELLKGRGLF^†^** | 3 (0.02) |
| **CCCGAACTCCCAAAGGAAAGAGGCCTGTTT** | substitution | **PELPKERGLF^†^** | 6 (0.04) |
| **CCCGAACTCCAAAAGGGAAGAGGCCTGTTT** | substitution | **PELQKGRGLF^†^** | 1 (0.01) |
| **CCCGAACTCCAACGGAAGGAAGGCCTGTTT** | substitution | **PELQRKEGLF^†^** | 1 (0.01) |
| **CCCGAACTCCCAAAGGGAAGAGACCTGTTT** | substitution | **PELPKGRDLF^†^** | 4 (0.03) |
| **CCCGAACTCCCAAAGAGAAGAGGCCTGTTT** | substitution | **PELPKRRGLF^†^** | 1 (0.01) |
| **CCCGAACTCCCAAAGGTAAGAGGCCTGTTT** | substitution | **PELPKVRGLF^†^** | 7 (0.05) |
| **CCCGAACTCCCAAGGGGAAGAGGCCTGTTT** | substitution | **PELPRGRGLF^†^** | 13 (0.10) |
| **CCCGAACTCCGAAAGGGAAGAGGCCTGTTT** | substitution | **PELRKGRGLF^†^** | 1 (0.01) |
| **CCCGAACTCCCAAAGGGAAGAGGCCCGTTT** | substitution | **PELPKGRGPF^†^** | 5 (0.04) |
| **CCCGAACGCCCAAAGGGAAGAGGCCTGTTT** | substitution | **PERPKGRGLF^†^** | 1 (0.01) |
| **CCCGAAACTCCC_AAGGGAAGAGGCCTGTTT** | substitution | **PETPKGRGLF^†^** | 2 (0.01) |
| **CCGGAACTCCCAAAGGGAAGAGGC_TGGTTT** | substitution | **PELPKGRGWF^†^** | 1 (0.01) |
| **CCCGAACCCCCAAAGGGAAGAGGCCTGTTT** | substitution | **PEPPKGRGLF^†^** | 16 (0.12) |
| **CCCGAACTCCCAAAGGGAAGAGGCCTGCTT** | substitution | **PELPKGRGLL^†^** | 47 (0.34) |
| **CCCGAACTCCCAAAGGGAAGAGGCCTGTCT** | substitution | **PELPKGRGLS^†^** | 3 (0.02) |
| **CCCGAACTCCCAAAGGGAAGAGGCCTGGTT** | substitution | **PELPKGRGLV^†^** | 49 (0.34) |
| **CCCGAACTCCCAAAGGGAAGAGGCCTGATTT** | +1 frameshift | **PELPKGRGLI** | 15 (0.11) |
| **CCCGAACTCCCAAAGGGAAGAGGCCATGTTT** | +1 frameshift | **PELPKGRGHV** | 6 (0.04) |
| **CCCGAACATCCCAAAGGGAAGAGGCCTGTTT** | +1 frameshift | **PEHPKGKRPV** | 5 (0.04) |
| **CCCGAACTCCCAAGGGGAAGAGGCCTGGTTT** | +1 frameshift | **PELPRGRGLV** | 1 (0.01) |
| **CCCGAACTCCCAAAGGGAAAGAGGCCTGTTT** | +1 frameshift | **PELPKGKRPV** | 11 (0.08) |
| **CCCGAACTCCCAAAGGGGAAGAGGCCTGTTT** | +1 frameshift | **PELPKGKRPV** | 68 (0.50) |
| **CCCGAACTCCCAAAGGGTAAGAGGCCTGTTT** | +1 frameshift | **PELPKGKRPV** | 3 (0.02) |
| **CCCGAACTTCCCAAAGGGAAGAGGCCTGTTT** | +1 frameshift | **PELPKGKRPV** | 2 (0.01) |
| **CCCGAACTCCCCAAAGGGAAGAGGCCTGTTT** | +1 frameshift | **PELPKGKRPV** | 27 (0.20) |
| **CCCGAACTCCCAAAAGGGAAGAGGCCTGTTT** | +1 frameshift | **PELPKGKRPV** | 8 (0.06) |
| **CCCGAACTCCCGAAAGGGAAGAGGCCTGTTT** | +1 frameshift | **PELPKGKRPV** | 11 (0.08) |
| **CCCGAACTCCCAAATGGGAAGAGGCCTGGTT** | +1 frameshift | **PELPNGKRPG** | 1 (0.01) |
| **CCCGAACTCCCAAATGGGAAGAGGCCTGTTT** | +1 frameshift | **PELPNGKRPV** | 10 (0.07) |
| **CCCGAACTACCCAAAGGGAAGAGGCCTGTTT** | +1 frameshift | **PELPKGKRPV** | 1 (0.01) |
| **CCCGAACTCCCAAAGGGAAGAGGGCCTGTTT** | +1 frameshift | **PELPKGRGPV** | 81 (0.59) |
| **CCCGAACTCCCAAAGGGAAGAGGCCGTGTTT** | +1 frameshift | **PELPKGRGRV** | 17 (0.12) |
| **CCCGAACTCCCAAAGGGAAGACGGCCTGTTT** | +1 frameshift | **PELPKGRRPV** | 36 (0.26) |
| **CCCGAACCCCCCAAAGGGAAGAGGCCTGTTT** | +1 frameshift | **PEPPKGKRPV** | 3 (0.02) |
| **CCCGAACCCCCAAAGGGAAGAGGGCCTGTTT** | +1 frameshift | **PEPPKGRGPV** | 1 (0.01) |
| **CCCGAACGTCCCAAAGGGAAGAGGCCTGTTT** | +1 frameshift | **PERPKGKRPV** | 1 (0.01) |
| **CCCGAAACTCCCAAGGGAAAGAGGCCTGTTT** | +1 frameshift | **PETPKGKRPV** | 31 (0.23) |
| **CCCGGAACTCCCAAAGGGAAGAGGCCTGTTT** | +1 frameshift | **PGTPKGKRPV** | 12 (0.09) |
| **CCCGAACTCCCAAAGGGAATGAGGCCTGTTT** | +1 frameshift | **PELPKGMRPV** | 6 (0.04) |
| **CCCGAACTCCCAAAGGGAAGTAGGCCTGTTT** | +1 frameshift | **PELPKGSRPV** | 15 (0.11) |
| **CCCGAACTCCCAAAGGGAACGAGGCCTGTTT** | +1 frameshift | **PELPKGTRPV** | 4 (0.03) |
| **CCCGAACTCCCAAAGGGAAGATGGCCTGTTT** | +1 frameshift | **PELPKGRWPV** | 3 (0.02) |
| **CCCGAACTCCCAAAGGGAATGAGGCCTCGTTT** | +2 frameshift | **PELPKGMRPR** | 1 (0.01) |
| **CCCGAACTCCCAAAGGGAAGAGGCTCCTGTTT** | +2 frameshift | **PELPKGRGSC** | 4 (0.03) |
| **CCCGAACTCCCAAAGGGAAGGAAGGCCTGTTT** | +2 frameshift | **PELPKGRKAC** | 3 (0.02) |
| **CCCGA_CTCCCCAAAGGGGAAGAGGGCCTGTTT** | +2 frameshift | **PDSPKGKRAC** | 1 (0.01) |
| **CCCGAACCTCCCAAAGGGAAGCAGGCCTGTTT** | +2 frameshift | **PEPPKGKQAC** | 1 (0.01) |
| **CCCGAACCTCCCAAAGGGAAGAGGGCCTGTTT** | +2 frameshift | **PEPPKGKRAC** | 1 (0.01) |
| **CCCGAACATCCCAAAGGGAAGAGGGCCTGTTT** | +2 frameshift | **PEHPKGKRAC** | 1 (0.01) |
| **CCCGAACTCCCCAAAAGGGAAGAGGCCTGTTT** | +2 frameshift | **PELPKREEAC** | 2 (0.01) |
| **CCCGAACTCCCAAAGGGGAAAGAGGCCTGTTT** | +2 frameshift | **PELPKGKEAC** | 2 (0.01) |
| **CCCGAACTCCCAAAGGGGAAGAAGGCCTGTTT** | +2 frameshift | **PELPKGKKAC** | 1 (0.01) |
| **CCCGAACTCCCAAAGGGTAAGATGGCCTGTTT** | +2 frameshift | **PELPKGKMAC** | 1 (0.01) |
| **CCCGAACTCCCAAAGGGAAAGCAGGCCTGTTT** | +2 frameshift | **PELPKGKQAC** | 1 (0.01) |
| **CCCGAACTCCCAAAGGGGAAGAGGGCCTGTTT** | +2 frameshift | **PELPKGKRAC** | 13 (0.10) |
| **CCCGAACTCCCGAAAGGGAAGAGGCCGTGTTT** | +2 frameshift | **PELPKGKRPC** | 2 (0.01) |
| **CCCGAACTCCCCAAAGGGAAGAGGCCTTGTTT** | +2 frameshift | **PELPKGKRPC** | 5 (0.04) |
| **CCCGAACTCCCAAAGGGGAAGAGGCCCTGTTT** | +2 frameshift | **PELPKGKRPC** | 1 (0.01) |
| **CCCGAAACTCCCAAAGGGAAGAAGGCCTGTTT** | +2 frameshift | **PETPKGKKAC** | 1 (0.01) |
| **CCCGAAACTCCCAAAGGGAAGAGGCCTTGTTT** | +2 frameshift | **PETPKGKRPC** | 2 (0.01) |
| **CCCGAAACTCCCAAAGGAAAGAGGCCTCGTTG** | +2 frameshift | **PETPKGKRPR** | 3 (0.02) |
| **CCCGAACTCCCAAACGGGAAGAGGCCGTGTTT** | +2 frameshift | **PELPNGKRPC** | 2 (0.01) |
| **CCCGAACTTCCCAAAGGGAAGAGGTCCTGTTT** | +2 frameshift | **PELPKGKRSC** | 1 (0.01) |
| **CCCGAACTCCCGAAAGGGAAGAGGACCTGTTT** | +2 frameshift | **PELPKGKRTC** | 1 (0.01) |
| **CCCGAAGCTCCCAAAGGGAAGAGGCCTCGTTT** | +2 frameshift | **PEAPKGKRPR** | 1 (0.01) |
| **CCCGAACTCCCAAAGGGAAGAGGCCTGGATTT** | +2 frameshift | **PELPKGRGLD** | 2 (0.01) |
| **CCCGAACTCCCAAAGGGAAGAGGTCCCTGTTT** | +2 frameshift | **PELPKGRGPC** | 5 (0.04) |
| **CCCGAACTCCCAAAGGGAAGAGGGCCTCGTTT** | +2 frameshift | **PELPKGRGPR** | 2 (0.01) |
| **CCCGAACTCCCAAAGGGAAGGAGGCCTTGTTT** | +2 frameshift | **PELPKGRRPC** | 2 (0.01) |
| **CCCGAACTCCCAAAGGGAAGACGGCCTCGTTT** | +2 frameshift | **PELPKGRRPR** | 4 (0.03) |
| **CCCGAACTCCCAAAGGGAAGAGTGGCCTGTTT** | +2 frameshift | **PELPKGRVAC** | 1 (0.01) |
| **CCCGAACTCCCAAAGGGAAGATGGTCCTGTTT** | +2 frameshift | **PELPKGRWSC** | 1 (0.01) |
| **CCCGAACTCCCAAAGGGAAGTAGGCCGTGTTT** | +2 frameshift | **PELPKGSRPC** | 2 (0.01) |
| **CCCGAAACTCCCAAAAGGGAAGAGGCCTGTTT** | +2 frameshift | **PETPKREEAC** | 2 (0.01) |
| **CCCGGAACTCCCAAAGGGAAAGAGGCCTGTTT** | +2 frameshift | **PGTPKGKEAC** | 1 (0.01) |
| **CCCGGAACTCCCAAAAGGGAAGAGGCCTGTTT** | +2 frameshift | **PGTPKREEAC** | 2 (0.01) |
| **CCCGGAACTCCCAAAGGGAAGAGGGCCTGTTT** | +2 frameshift | **PGTPKGKRAC** | 1 (0.01) |
| **CCCGGACTCCCAAAGGGGAAGAGGCCCTGTTT** | +2 frameshift | **PGLPKGKRPC** | 1 (0.01) |
| **CCCGAAGCTCCCAAAGGGAAGAGGCCTGATTT** | +2 frameshift | **PEAPKGKRPD** | 1 (0.01) |
| **CCCGAACTCCCAAAGGGAACGAGGCCTTGTTT** | +2 frameshift | **PELPKGTRPC** | 3 (0.02) |
| **CCCGAAGCTCCCAAAGGGAAGAGGCCTGATTT** | +2 frameshift | **PEAPKGKRPD** | 1 (0.01) |
| **CCCGAACTCCCGAAAGGGAAGACAGGCCTGTTT** | insertion/substitution | **PELPKGKTGLF^†^** | 1 (0.01) |
| **CCCGAAGCTCCCAAAGGGAAGAGGCCGTGGTTT** | insertion/substitution | **PEAPKGKRPWF^†^** | 1 (0.01) |
| **CCCGAAGCTCCCAAACGGGAAGAGGCCTCGTTT** | insertion/substitution | **PEAPKREEASF^†^** | 1 (0.01) |
| **CCCGAACATCCCAAACGGGAAAGAGGCCTGTTT** | insertion/substitution | **PEHPKRERGLF^†^** | 1 (0.01) |
| **CCCGAACTCCCAAAGGGGAAGAGGGCCGTGTTT** | insertion/substitution | **PELPKGKRAVF^†^** | 1 (0.01) |
| **CCCGAACTCCCAAAGGGGAAAGAGGCCTTGTTT** | insertion/substitution | **PELPKGKEALF^†^** | 1 (0.01) |
| **CCCGAACTCCCAAAGGGAAGATGGCCTCGATTT** | insertion/substitution | **PELPKGRWPRF^†^** | 1 (0.01) |
| **CCCGAACTCCCGAAAAGGGAAGAGGTCCTGTTT** | insertion/substitution | **PELPKREEVLF^†^** | 1 (0.01) |
| **CCCGAACTCCCAAAGGGAACGAGGCACCTGTTT** | insertion/substitution | **PELPKGTRHLF^†^** | 1 (0.01) |
| **CCCGAACTCCCAAAGGGAAGAGGCCCTCGATTT** | insertion/substitution | **PELPKGRGPRF^†^** | 2 (0.01) |
| **CCCGAATCTCCCAAAGGGAAGAAGGCCTTGTTT** | insertion/substitution | **PESPKGKKALF^†^** | 1 (0.01) |
| **CCCGAAACTCCCCAAAAGGGAAGAGGCCTGTTT** | insertion/substitution | **PETPQKGRGLF^†^** | 1 (0.01) |
| **CCCGAAACTCCCAAAGGGAAGGAGGGCCTGTTT** | insertion/substitution | **PETPKGKEGLF^†^** | 1 (0.01) |
| **CCCGAACTTCCCAAAGGGAGGAGGGCCCTGTTT** | insertion/substitution | **PELPKGRRALF^†^** | 1 (0.01) |
| **CCCGGAAACTCCCGAAAGGGAAGAGGCCTGTTT** | insertion/substitution | **PGNSRKGRGLF^†^** | 1 (0.01) |
| **CCCGGAACTCCCAAAGGGAAGAGGTCCATGTTT** | insertion/substitution | **PGTPKGKRSMF^†^** | 1 (0.01) |
| **CCCGAACTCCCAAAGGGAAAGGAGGCACCTGTTT** | +1 frameshift | **PELPKGKEAPV** | 1 (0.01) |
| **CCCGAACTCCCAAAGGGGAAAGAAGGCCTTGTTT** | +1 frameshift | **PELPKGKEGLV** | 1 (0.01) |
| **CCCGAACTCCCAAAAGGGGAAAGAGGCCCTGTTT** | +1 frameshift | **PELPKGERGPV** | 1 (0.01) |
| **CCCGAACTCCCGAAAGGGAAGAGGGCCTTGGTTT** | +1 frameshift | **PELPKGKRALV** | 1 (0.01) |
| **CCCGAACTCCCCAAAAGGGGAAGAAGGCCTGTTT** | +1 frameshift | **PELPKRGRRPV** | 4 (0.03) |
| **CCCGAACTCCCAAAGGGAAGAAGGTTACTGATTT** | +1 frameshift | **PELPKGRRLLI** | 1 (0.01) |
| **CCCGAACTTCCCCAAAGGGGAAGAGGGCCTGTTT** | +1 frameshift | **PELPQRGRGPV** | 1 (0.01) |
| **CCCGAACTCCCCAAAAGGGAAGAGGGCCGTGTTT** | +1 frameshift | **PELPKREEGRV** | 1 (0.01) |
| **CCCGAAGCTCCCAAAGGGAAGGAGGGCCATGTTT** | +1 frameshift | **PEAPKGKEGHV** | 1 (0.01) |
| **CCCGAACCTCCCAAAGGGAAGACTGGGCCTGTTT** | +1 frameshift | **PEPPKGKTGPV** | 1 (0.01) |
| **CCCGAACTCCCAAAGGGAAGATGGCTCCTTGTTT** | +1 frameshift | **PELPKGRWLLV** | 1 (0.01) |
| **CCCGAAACTCCCCAAAGGGGAAAGAGGCCTGTTT** | +1 frameshift | **PETPQRGKRPV** | 1 (0.01) |
| **CCCGGAACTCCCAAAGGGAAGAAGGTCCATGTTT** | +1 frameshift | **PGTPKGKKVHV** | 1 (0.01) |
| **CCCGAAACTCCCCAAAAGGGGAAAGAGGCCTGTTT** | +2 frameshift | **PETPQKGKEAC** | 1 (0.01) |
| **CCCGAATCTCCCCAAAGGGGAAGAGGGCCTGTTTT** | +2 frameshift | **PESPQRGRGPV** | 1 (0.01) |
| **CCCGAACTTCCCAGAAAGGGAAAGAGGCCATGTTT** | +2 frameshift | **PELPRKGKRPC** | 1 (0.01) |
| **CCCGAAGCTCCCAAAGGGAAGGAGTGGGCCTGTTT** | +2 frameshift | **PEAPKGKEWAC** | 1 (0.01) |
| **CCCGAAACTCCCCAAAAGGGAAAGAGGCCCTGTTT** | +2 frameshift | **PETPQKGKRPC** | 1 (0.01) |
| **CCCGAAACTCCCCAAAAGGGGAAGGAGGCCTTGTTT** | insertion/substitution | **PETPQKGKEALF^†^** | 1 (0.01) |
| **CCCGAAACTCCCCAAAAGGGGAAAGAGGGCCTGTTT** | insertion/substitution | **PETPQKGKEGLF^†^** | 1 (0.01) |
| **CCCGAACTTCCCAAAGGGAAGAAGGGCCGATGGTTT** | insertion/substitution | **PELPKGKKGRWF^†^** | 1 (0.01) |
|  |  | Total no. reads: | 13681 |
|  |  | No. variants: | 134 [0.01] |

1. **H7N1 Passage 17**

| **HA_0_ cDNA nucleotide sequence^#^** | **Main mutational**  **effect** | **Translated**  **amino acids** | **No. of reads**  **(percentage)** |
| --- | --- | --- | --- |
| **CCCGAACTCCCAAAGGGAAGAGGCCTGTTT**^‡^ |  | **PELPKGRGLF^†^** | 7079 (97.24) |
| **CCCGAACTCCCAA_GGGA_GAGGCCTGTTT** | +2 frameshift | **PELPRERPV** | 1 (0.01) |
| **CCCGAACTCC__AAGGGAAGAGGCCTGTTT** | +2 frameshift | **PELQGKRPV** | 1 (0.01) |
| **CCCGAACTCCCAA_GGGAAGAGGCCTGTTT** | +1 frameshift | **PELPKGKRPV** | 2 (0.03) |
| **CCCGAACTCC_AAGGGAAGAGGCCGTGTTT** | +1 frameshift | **PELQGKRPC** | 1 (0.01) |
| **CCCGAACTCCCAA_GGGGAAGAG_CCTGTTT** | +1 frameshift | **PELPRGRAC** | 1 (0.01) |
| **CCCGAACTCCCAAAGGGAAAAGGCCTGTTT** | substitution | **PELPKGKGLF^†^** | 1 (0.01) |
| **CCCGAACTCCCAAAGGGAAGATGCCTGTTT** | substitution | **PELPKGRCLF^†^** | 1 (0.01) |
| **CCCGAACTCCCAAAGGGAAGAGGCCTGCTT** | substitution | **PELPKGRGLL^†^** | 3 (0.04) |
| **CCCGAACTCCCAAAGGGAAGAGGCCCGTTT** | substitution | **PELPKGRGPF^†^** | 1 (0.01) |
| **CCCGAACTCTCAAAGGGAAGAGGCCTGTTT** | substitution | **PELSKGRGLF^†^** | 2 (0.03) |
| **CCCGGACTCCCAAAGGGAAGAGGCCTGTTT** | substitution | **PGLPKGRGLF^†^** | 2 (0.03) |
| **CCCGAACTCCCAAGGGGAAGAGGCCTGTTT** | substitution | **PELPRGRGLF^†^** | 8 (0.12) |
| **CCCGAACTCCCAAAGAGAGGAGGCCTGTTT** | substitution | **PELPKRGGLF^†^** | 1 (0.01) |
| **CCCGAACTCCCAAAGGGAAGAGGCCTGGTTT** | +1 frameshift | **PELPKGRGLV** | 6 (0.08) |
| **CCCGAACTCCCAAAGGGAAGAGGCCTCGTTT** | +1 frameshift | **PELPKGRGLV** | 6 (0.08) |
| **CCCGAACTCCCAAAGGGAAGAGGCCTTGTTT** | +1 frameshift | **PELPKGRGLV** | 4 (0.05) |
| **CCCGAACTCCCAAAGGGAAGTAGGCCTGTTT** | +1 frameshift | **PELPKGSRPV** | 3 (0.04) |
| **CCCGAACTCCCAAAGGAAAGAAGGCCTGTTT** | +1 frameshift | **PELPKERRPV** | 1 (0.01) |
| **CCCGAACTCCCAAAGGGAAAGAGGCCTGTTT** | +1 frameshift | **PELPKGKRPV** | 1 (0.01) |
| **CCCGAACTCCCAAAGGGAATGAGGCCTGTTT** | +1 frameshift | **PELPKGMRPV** | 5 (0.07) |
| **CCCGAACTCCCCAAAGGGAAGAGGCCTGTTT** | +1 frameshift | **PELPKGKRPV** | 4 (0.05) |
| **CCCGAACTCCCGAAAGGGAAGAGGCCTGTTT** | +1 frameshift | **PELPKGKRPV** | 5 (0.07) |
| **CCCGAACTCCCAAAAGGGAAGAGGCCTGTTT** | +1 frameshift | **PELPKGKRPV** | 2 (0.03) |
| **CCCGAACTCCCAAAGGGTAAGAGGCCTGTTT** | +1 frameshift | **PELPKGKRPV** | 2 (0.03) |
| **CCCGAACTTCCCAAAGGGAAGAGGCCTGTTT** | +1 frameshift | **PELPKGKRPV** | 1 (0.01) |
| **CCCGAACTCCCAAAGGGGAAGAGGCCTGTTT** | +1 frameshift | **PELPKGKRPV** | 26 (0.36) |
| **CCCGAACTCCCAAAGGGAAGGAGGCCTGTTT** | +1 frameshift | **PELPKGKRPV** | 5 (0.07) |
| **CCCGAACATCCCAAAGGGAAGAGGCCTGTTT** | +1 frameshift | **PEHPKGKRPV** | 2 (0.03) |
| **CCCGAACTCCCAAAGGGAAGAGGGCCTGTTT** | +1 frameshift | **PELPKGRGPV** | 14 (0.19) |
| **CCCGAACTCCCAAAGGGAAGAGGCCGTGTTT** | +1 frameshift | **PELPKGRGRV** | 5 (0.07) |
| **CCCGAACTCCCAAAGGGAAGAGGACCTGTTT** | +1 frameshift | **PELPKGRGPV** | 3 (0.04) |
| **CCCGAACTCCCAAAGGGAAGAGGTCCTGTTT** | +1 frameshift | **PELPKGRGPV** | 5 (0.07) |
| **CCCGAACTCCCAAAGGGAAGAGGCCCTGTTT** | +1 frameshift | **PELPKGRGPV** | 1 (0.01) |
| **CCCGAAGCTCCCAAAGGGAAGAGGCCTGTTT** | +1 frameshift | **PEAPKGKRPV** | 8 (0.12) |
| **CCCGAACTCCCAAAGGGAAGATGGCCTGTTT** | +1 frameshift | **PELPKGRWPV** | 3 (0.04) |
| **CCCGAACTCCCAAAGGGAAGTAGGCCTGTTT** | +1 frameshift | **PELPKGSRPV** | 5 (0.07) |
| **CCCGAACTCCCAAAGGGAAGCAGGCCTGTTT** | +1 frameshift | **PELPKGSRPV** | 1 (0.01) |
| **CCCGAACTCCCAAAGGGAACGAGGCCTGTTT** | +1 frameshift | **PELPKGTRPV** | 3 (0.04) |
| **CCCGAACTCCCAAAGAGAATGAGGCCTGTTT** | +1 frameshift | **PELPKRMRPV** | 1 (0.01) |
| **CCCGAACGTCCCAAAGGGAAGAGGCCTGTTT** | +1 frameshift | **PERPKGKRPV** | 2 (0.03) |
| **CCCGAATCTCCCAAAGGGAAGAGGCCTGTTT** | +1 frameshift | **PESPKGKRPV** | 2 (0.03) |
| **CCCGGAACTCCCAAAGGGAAGAGGCCTGTTT** | +1 frameshift | **PGTPKGKRPV** | 1 (0.01) |
| **CCCGAAACTCCCAAAGGGAAGAGGCCTGTTT** | +1 frameshift | **PETPKGKRPV** | 8 (0.12) |
| **CCCGAACTCCCCAAAGGGAAGAGGCCTCGTTT** | +2 frameshift | **PELPKGKRPR** | 2 (0.03) |
| **CCCGAACTCCCAAAGGGAAGAGGGCCGTGTTT** | +2 frameshift | **PELPKGRGPC** | 1 (0.01) |
| **CCCGAACTCCCAAAGGGAAGAGGACCTGATTT** | +2 frameshift | **PELPKGRGPD** | 1 (0.01) |
| **CCCGAACTCCCCAAAGGGAAGAAGGCCTGTTT** | +2 frameshift | **PELPKGKKAC** | 5 (0.07) |
| **CCCGAACTCCCAAAGGGAAGAGGCCGTCGTTT** | +2 frameshift | **PELPKGRGRR** | 1 (0.01) |
| **CCCGAACTCCCAAAGGGAAGTAAGGCCTGTTT** | +2 frameshift | **PELPKGSKAC** | 1 (0.01) |
| **CCCGAACTCCCAAAGGGAAGACGGCCGTGTTT** | +2 frameshift | **PELPKGRRPC** | 2 (0.03) |
| **CCCGAACTCCCAAAGGGAAGGAGGCCTCGTTT** | +2 frameshift | **PELPKGRRPR** | 1 (0.01) |
| **CCCGAAGCTCCCAAAGGGAAGAAGGCCTGTTT** | +2 frameshift | **PEAPKGKKAC** | 1 (0.01) |
| **CCCGAAGCTCCCAAAGGGAAGAGGCCTCGTTT** | +2 frameshift | **PEAPKGKRPR** | 1 (0.01) |
| **CCCAAAACTCCCAAAGGGAAGAGGTCCTGTTT** | +2 frameshift | **PKTPKGKRSC** | 1 (0.01) |
| **CCCGAACATCCCAAAGGGAAGAGGCCGTGTTT** | +2 frameshift | **PEHPKGKRPC** | 2 (0.03) |
| **CCCGAATCCCCAAAGGGAAGTAAGGCCTGTTT** | +2 frameshift | **PESPKGSKAC** | 1 (0.01) |
| **CCCGAAACTCCCAAAGGGAAGAGGCCGTGTTT** | +2 frameshift | **PETPKGKRPC** | 1 (0.01) |
| **CCCGAATCTCCCAAAGGGAAGAGGCCATGTTT** | +2 frameshift | **PESPKGKRPC** | 1 (0.01) |
| **CCCGAACCTCCCAAAGGGAAGAGGGCCTGTTT** | +2 frameshift | **PEPPKGKRAC** | 1 (0.01) |
| **CCCGAACTCCCCAAAAGGGAAGAGGCCTGTTT** | +2 frameshift | **PELPKREEAC** | 1 (0.01) |
| **CCCGAACTCCCAAAGGGAACGAGGCCGTGTTT** | +2 frameshift | **PELPKGTRPC** | 1 (0.01) |
| **CCCGAACTCCCAAACGGGAAGAGGCCTCGTTT** | +2 frameshift | **PELPNGKRPR** | 1 (0.01) |
| **CCCGAACTCCCAAAGGGAAGAACGGCCCTGTTT** | insertion/substitution | **PELPKGRTALF^†^** | 1 (0.01) |
| **CCCGAACTCCCAAAGGGAACGAGGGCCTGGTTT** | insertion/substitution | **PELPKGTRAWF^†^** | 1 (0.01) |
| **CCCGGAACTCCCAAAAGGGAAGAGGGCCTGTTT** | insertion/substitution | **PGTPKREEGLF^†^** | 1 (0.01) |
| **CCCGGAACTCCCAAAGGGAAGGAGGCCTGGTTT** | insertion/substitution | **PGTPKGKEAWF^†^** | 1 (0.01) |
| **CCCGGAACTCCCAAAGGGAAAGGAGGCCTGTTT** | insertion/substitution | **PGTPKGKGGLF^†^** | 1 (0.01) |
| **CCCGAAACTCCCAAACGGGAAGAGGGCCTGTTT** | insertion/substitution | **PETPKREEGLF^†^** | 1 (0.01) |
| **CCCGCCGAACTCCCAAAGGGAAGAGGCCTGTTT** | insertion/substitution | **PAELPKGRGLF^†^** | 1 (0.01) |
| **CCCGGAACTCCCAAATGGGAAGTAGGCCGTGTTT** | +1 frameshift | **PGTPKWEVGRV** | 1 (0.01) |
| **CCCGGAACTCCCCAAAGGGGAAGAGGGCCTGTTT** | +1 frameshift | **PGTPQRGRGPV** | 1 (0.01) |
| **CCCGGAACTCCCCGAAAGGGAAGGAGGCCTGTTT** | +1 frameshift | **PGTPRKGRRPV** | 2 (0.03) |
| **CCCGAAGCTCCCAAACGGGAAGAGGGCCTCGTTT** | +1 frameshift | **PEAPKREEGLV** | 1 (0.01) |
| **CCCGAACATCCCAAACGGGAAGCAGGACCTGTTT** | +1 frameshift | **PEHPKREAGPV** | 1 (0.01) |
| **CCCGAACTCCCCGAAACGGGAAGGAGGCCTGTTT** | +1 frameshift | **PELPETGRRPV** | 1 (0.01) |
| **CCCGAACCTCCCCAAAGGGAACGACGGCCGTGGTTT** | insertion/substitution | **PEPPQRERRPWF^†^** | 1 (0.01) |
|  |  | Total no. reads: | 7280 |
|  |  | No. variants: | 77 [0.01] |

^#^Insertions, deletions and substitutions in relation to the conventional low pathogenic sequence^‡^ are underlined; stop codons are indicated by “_*_”.

^†^In-frame HA_2_

[ ] total number of variants: total number of HACS read ratio
